# Digging into the Solubility Factor in Cancer Diagnosis: A Case of Soluble CD44 Protein

**DOI:** 10.3390/bios15120796

**Published:** 2025-12-04

**Authors:** Zhuldyz Myrkhiyeva, Marzhan Nurlankyzy, Kulzhan Berikkhanova, Zhanas Baimagambet, Aidana Bissen, Nurzhan Bikhanov, Christabel K. L. Tan, Daniele Tosi, Zhannat Ashikbayeva, Aliya Bekmurzayeva

**Affiliations:** 1 Laboratory of Biosensors and Bioinstruments, Center for Life Sciences, National Laboratory Astana, Nazarbayev University, Astana 010000, Kazakhstan; zhuldyz.myrkhiyeva@nu.edu.kz (Z.M.); marzhan.nurlankyzy@nu.edu.kz (M.N.); kberikkhanova@nu.edu.kz (K.B.); daniele.tosi@nu.edu.kz (D.T.); 2 School of Sciences and Humanities, Nazarbayev University, Astana 010000, Kazakhstan; 3 School of Medicine, Nazarbayev University, Astana 010000, Kazakhstan; zhanas.baimagambet@nu.edu.kz; 4 University Medical Center, Nazarbayev University, Astana 010000, Kazakhstan; nbikhanov@mail.ru; 5 School of Engineering and Digital Sciences, Nazarbayev University, Astana 010000, Kazakhstan; aidana.bissen@nu.edu.kz; 6 School of Physics, Engineering and Computer Science, College Lane, University of Hertfordshire, Hatfield AL10 9AB, UK; c.k.l.tan@herts.ac.uk

**Keywords:** soluble CD44 protein, CD44 molecule, biological fluids, cancer diagnosis, prognostic biomarker, protein detection methods

## Abstract

The detection of soluble proteins in biological fluids, as a form of liquid biopsy, is a promising tool for cancer diagnosis and prognosis, as it is less invasive than traditional diagnostic methods. CD44 is one of the most recognized markers of cancer stem cells, a small subset of cells responsible for cancer initiation, progression, and metastasis. Given the importance of CD44 as a cancer biomarker, several review articles explore the diagnostic and therapeutic value of cell-surface CD44. In addition to being a membrane-anchored protein, CD44 is also shed from the cell surface and can be found in various biological fluids. However, the role of soluble CD44 in cancer has not been comprehensively discussed in recent reviews. Measuring soluble CD44 in various biological liquids can provide a practical and valuable tool for cancer diagnosis and treatment monitoring. Therefore, this review comprehensively discusses the role of soluble CD44 as a marker in various cancer types, including serum, saliva, urine, and other fluids. In particular, its role as an early cancer biomarker and as a predictive and prognostic biomarker in several cancers is discussed. This work also provides an overview of a wide range of analytical techniques used to detect soluble CD44. The value of cells expressing CD44 versus soluble CD44 as a biomarker is also compared. The review concludes with a perspective on future directions, emphasizing the shift toward non-invasive analytical methods and the need for standardization of detection, including multiple biomarkers during evaluation, to improve the accuracy of cancer diagnosis.

## 1. Introduction

In recent years, we have witnessed a shift from histopathological techniques to molecular methods for diagnostics [1]. The molecular processes underlying cancer diagnosis help provide a more personalized strategy. Compared with DNA analysis, analysis of protein biomarkers is particularly valuable, as they serve as more direct indicators of the normal state versus cancer [2]. The detection of soluble proteins in biological fluids represents one modality of liquid biopsy and is a promising tool for the early diagnosis and continuous monitoring of cancer [3]; this method is less invasive than traditional methods [4]. Moreover, for some types of cancer, identifying high-risk precancerous states and further applying necessary treatment could be essential measures to reduce cancer mortality and morbidity and lower cancer treatment costs [1].

In the late 1990s, CD44 protein was considered one of the most promising candidate biomarkers for early cancer diagnosis [5]. Since then, its role in different cancer types has been widely studied, and it has been coined a multifunctional mediator of cancer progression [6,7]. Given the importance of CD44 as a cancer biomarker, it has been reviewed in several articles, many of which discuss its role as a biomarker and therapeutic target [8,9,10]. Review papers featuring developments related to the therapeutic implications of CD44 [9,11,12,13], its role in chemoresistance [14] and metastasis [7,15], its potential role in diagnosis [10,16,17], and its role in glioblastoma multiforme [18] have been published. An earlier excellent review from 2003 [19] is also available. More recent reviews discuss the role of its tissue-expressed form in cancer [20,21], including nanobiosensing approaches for early cancer detection in CD44-expressing cells [22], a particular form of cancer [23], or the intracellular domain of CD44 [22].

The year 2025 marks 42 years since the first discovery of soluble CD44 (solCD44) in serum [23]. However, the role of cell-surface CD44 in cancer has been studied more than its soluble form [24]. Proteolytic cleavage of membrane-bound CD44 generates a soluble form of CD44 that enters biological fluids and reflects dynamic tumor-associated processes such as proliferation, invasion, and extracellular matrix remodeling [25]. Key characteristics include its ability to indicate tumor burden and metastasis across multiple cancer types, including gastric, colon, and head and neck cancer [26]. Indeed, all of the abovementioned reviews also reflect this, focusing on the cell-surface CD44 marker rather than the soluble form. To the best of our knowledge, there are currently no comprehensive reviews that encompass the full range of CD44 protein detection methods. While there is an excellent section in a book chapter on the CD44 protein [27] and a short review [28] that provide valuable insight, they need to be updated, especially given increased knowledge about solCD44. A recent review discussed the role of CD44-expressing cells in bladder cancer and positioned CD44 as a promising biomarker and therapeutic target for noninvasive diagnosis and innovative treatments [29]. We analyzed the distribution of systematic reviews and meta-analyses on cell-expressed CD44 from 1995 to 2022, and the data indicate a research emphasis on gastric and breast cancers, which together accounted for more than 1/3 of all studies (Figure S1). Cancers of the head and neck, pancreas, ovaries, bone (osteosarcoma), and central nervous system (gliomas) were the most widely studied, indicating overall but unequal interest in different tumor types.

To date, no reviews have focused solely on solCD44 as a cancer biomarker. Specifically, this work focuses on less studied forms solCD44, including proteolytically shed protein and exosome-associated form of CD44. Given that protein detection in biological fluids offers a more straightforward diagnostic approach than cell- or tissue-based techniques, this review discusses the significance of solCD44 as a cancer biomarker across various biological fluids. The role of solCD44 as a diagnostic, prognostic, and predictive biomarker of cancer will be discussed, as will its role in other biological fluids, such as saliva, urine, and others. The review will also provide an overview of solCD44, its ligands, and the assays used for its detection, and will compare solCD44 with the cell-expressed form of the protein. The graphical abstract shows the different aspects covered in this review. The soluble forms of the CD44 protein and its isoforms are named differently in the literature, as shown in Table 1. To avoid misunderstanding, three unified names are used throughout this review.

## 2. Shedding of the Soluble Form of CD44 from the Cell

CD44 is encoded by a single gene located on chromosome 11: the Lutheran inhibitory gene In(Lu) [23]. CD44 expression in normal epithelium is mainly in the basal and suprabasal regions, but CD44 can migrate into the superficial layers, suggesting its role in early carcinogenesis [46]. CD44 is overexpressed during the initial stages of tumor development, migration, proliferation, and metastasis [45]. There is also evidence of cell-surface CD44 enrichment in recurring tumors, which occurs during CD44 cleavage and release by metalloproteinases [46].

The transmembrane protein CD44 has a standard form (CD44s), and a family of splice variants, CD44v1–CD44v10, are produced by alternative splicing [47]. The CD44 protein consists of two sets of exons. Exons 1 through 5 and 16 through 20 create the standard CD44. Exons 6–15 (referred to as variable exons v1–v10) can be alternatively spliced and inserted between exons 5 and 16 [48]. CD44s is the smallest and most common isoform of CD44, at 85–95 kDa, and lacks the variable region generated by alternative splicing. It is expressed mainly on hematopoietic cells, particularly leukocytes, and helps immune cells traffic and adhere [47]. Other isoforms, such as CD44v8–CD44v10, are found in some epithelial cells. In normal tissue, the expression of CD44 isoforms with combinations of other variant exons is less prevalent. Still, in hematopoietic cells, particularly peripheral blood mononuclear cells and reactive lymph node cells, variant isoforms are expressed. The variant forms of CD44 are expressed in restricted locations; CD44v6 and CD44v9 are expressed on T lymphocytes and leucocytes, whereas CD44v8, CD44v9, and CD44v10 are expressed on epithelial cells and keratinocytes [47]. There is evidence that the breast and pancreatic ducts express CD44v6 isoforms, whereas the v4-containing isoform is expressed in the normal urothelium. Some cell types, such as hepatocytes, pancreatic acinar cells, and tubules of the kidney and pancreas, do not express any CD44 isoforms [5]. The expression of specific CD44 isoforms is correlated with the corresponding cancer type [49] (see Supplementary document for a more detailed discussion).

CD44 cleavage, also known as shedding, is a key process in cancer progression and contributes to tumor invasion, metastasis, and cellular signaling. The shedding process releases sCD44 into the serum, and its level often correlates with tumor burden in cancers such as gastric, colon, and non-Hodgkin lymphoma, and typically decreases after tumor removal or chemotherapy [26,49]. This process is initiated by proteolytic cleavage of the CD44 ectodomain, predominantly mediated by ADAM10, ADAM17, and MMP14 (MT1-MMP), and driven by stimuli such as Ca^2+^ influx, protein kinase C activation, and small GTPases [50]. These cleavages modulate cell adhesion, migration, and invasion by reducing CD44′s adhesive properties and enhancing its role in cell mobility within the extracellular matrix.

CD44 ectodomain shedding often occurs at the leading edge of migrating tumor cells, where it forms a complex with MMP14 to facilitate cytoskeletal interactions and tumor invasion [50]. This process is further supported by diverse triggers, including extracellular ATP, which induces CD44 shedding via the P2X7 receptor in macrophage-like cells, and by Ras oncoproteins and growth factors such as epidermal growth factor [51]. The ECM interaction is further refined by other proteases, such as meprin β, which are predominantly active in tissues such as the gastrointestinal tract, kidneys, and immune cells. Together, these mechanisms position CD44 as a dynamic regulator of tumor cell behavior [51].

Following ectodomain shedding, CD44 undergoes intramembrane proteolysis mediated by γ-secretase. This cleavage generates the intracellular domain of CD44, which translocates to the nucleus to activate transcription factors, including tetradecanoylphorbol acetate-responsive elements and the transcriptional coactivator CBP/p300 [52]. These processes drive the expression of genes involved in cell survival, proliferation, and migration, establishing a feedback loop that amplifies CD44′s role in tumor progression and metastasis [52].

Early studies revealed that at least five prostate cancer cell lines released the CD44 protein into the media by shedding it when grown in serum-free media. Cell surface expression decreases 3–7-fold, whereas circulatory protein levels increase 6–20-fold [41]. Studies have shown that CD44 cleavage is widespread across various malignancies, with ectodomain cleavage detected in 58% of gliomas, 67% of breast carcinomas, 90% of colon carcinomas, and other cancers, including non-small cell lung carcinoma and ovarian carcinoma. These findings suggest a direct role for CD44 shedding in tumor malignancy and metastasis. In particular, the hyaluronic acid-CD44 axis in the tumor microenvironment promotes epithelial-to-mesenchymal transition, thereby further enhancing cancer cell invasion and migration [53].

Therapeutic approaches targeting CD44 cleavage and its associated pathways are emerging as promising strategies in cancer treatment. Inhibiting proteases such as ADAM10 or γ-secretase can reduce CD44 shedding, thereby inhibiting its contribution to tumor growth and metastasis. Similarly, interventions such as trastuzumab, which inhibits ErbB-2-mediated shedding, have potential in controlling the impact of CD44. By disrupting CD44 turnover at the cell surface, these therapies may effectively reduce cancer cell migration and metastatic potential, offering new hope for improving patient outcomes [25].

## 3. The Role of Soluble CD44 Protein as a Cancer Biomarker

### 3.1. SolCD44 as a Diagnostic Biomarker for Early Cancer Detection and Classification of Cancer into Subgroups

Early identification of malignancies greatly enhances treatment outcomes and survival rates, underscoring the clinical relevance of biomarkers. Salivary solCD44 has strong diagnostic potential for head and neck squamous cell carcinoma (HNSCC). In a study involving 102 HNSCC patients and 69 controls, solCD44 distinguished malignant cases from benign cases with 62% sensitivity and 88% specificity [54]. Franzmann and Donovan [55] also validated OncAlert™, a point-of-care salivary test for oral and oropharyngeal cancer, further supporting its clinical utility. The OncAlert™ test was effectively used in early screening, and the total salivary protein also emerged as a complementary early marker of oral cancer. In potentially malignant oral disorders such as leukoplakia and erythroplakia, elevated salivary solCD44 levels were correlated with disease presence and transformation risk [45]. Another study (2011) [44] reported that the concentration of solCD44 in patients with HNSCC was significantly elevated compared with that in healthy individuals.

The potential role of solCD44 in non-Hodgkin’s lymphoma (NHL), breast, and colorectal carcinoma has been investigated [56], and its impact on disease prognosis has been determined. In this study, the total level of solCD44 in the serum of patients with non-Hodgkin lymphoma was higher than that in patients with breast or colorectal cancer, suggesting the potential diagnostic value of solCD44 in NHL.

Another group conducted a study on the expression and shedding of CD44 variant isoforms in patients with gynecologic malignancies. It evaluated the presence of CD44 isoforms in ascitic fluid and serum samples from patients with gynecologic cancers. This study included patients at various stages of gynecologic malignancies and analyzed the occurrence of solCD44v4/5 and solCD44v6 in tumor cells, serum, and ascitic fluid. The findings revealed that ovarian tumor cells expressed significant levels of CD44, particularly the v4/5 and v6 isoforms. CD44 isoforms were detectable in the serum of 6 of 8 cancer patients and in 12 of 16 ascitic fluid samples. Notably, all CD44-positive sera expressed detectable levels of variant CD44, with solCD44v6 being present in all positive samples. In ascitic fluid, CD44 is associated mainly with membrane fragments (vesicles). The study concluded that the differential expression and shedding of CD44 isoforms might play crucial roles in tumor metastasis and immune evasion [57]. In patients with cervical cancer, solCD44 levels were significantly elevated compared with those in controls (664.80 vs. 275.19 ng/mL), with even higher concentrations in postmenopausal patients than in premenopausal patients (658.88 vs. 593.69 ng/mL) [1]. Notably, solCD44v6 was detectable, whereas the standard and v5 isoforms were not detectable, suggesting isoform-specific relevance.

Identifying cancer molecular subtypes is crucial for guiding targeted therapy decisions, predicting treatment response, and improving patient outcomes. Because cancers of the exact tissue origin can differ significantly in their genetic, phenotypic, and immunologic profiles, biomarkers that help distinguish these subtypes are essential for the development of personalized medicine strategies. SolCD44, particularly its isoforms, has shown potential to reflect tumor aggressiveness and subtype characteristics in some types of cancers. Elevated serum levels of solCD44v5 and solCD44v6 were detected in node-positive breast cancer patients compared with node-negative patients and healthy controls, whereas solCD44std levels remained unchanged [58]. Urinary CD44 levels were positively correlated with high-grade bladder cancer compared with both control subjects and patients with low-grade tumors [59]. This study highlights solCD44 as a noninvasive biomarker that could aid in distinguishing aggressive bladder cancer subtypes and informing treatment decisions.

SolCD44std and solCD44v6 were measured in pleural fluid to identify whether malignant pleural effusion was spreading [60]. SolCD44v6 was found mainly in the pleural fluid of nonmesothelioma malignancies. solCD44v6 was also identified as a potential biomarker for differential diagnosis of pleural fluid malignancy.

Another set of studies revealed that solCD44 is less reliable as a diagnostic cancer biomarker. When solCD44 levels were evaluated across vocal fold lesion types, interestingly, the highest concentrations were observed in benign lesions and low-grade dysplasia, with a decline in high-grade and invasive cancers [61]. This inverse trend suggests that solCD44 may not reliably indicate malignancy risk in vocal fold pathology. In HPV-related oropharyngeal squamous cell carcinoma (OPSCC), a comparative analysis of salivary biomarkers indicated that DNA detection (particularly that of the HPV E6/E7 genes) was the most accurate. While solCD44 and other protein markers have shown potential, they are less reliable than genetic markers for early OPSCC diagnosis [62]. An earlier work from 1999 studied the concentration of solCD44v6 in HNSCC [63] and found no significant difference between patients and healthy controls, whereas a more recent study from 2011 [44] analyzed CD44 in all its forms and reported increased levels in cancer patients and demonstrated a correlation. A study by Kainz et al. showed that serum concentrations of solCD44std and solCD44v5 were not significantly associated with whether cervical cancer was present or absent [31]. In non-small cell lung cancer (NSCLC), standard solCD44 and isoform 6 levels did not differ between cancer patients and the control group (benign lung disease). In contrast, isoform 6 levels in squamous cell carcinoma patients were significantly higher than in the control group. Neither protein form was associated with cancer stage or metastasis in NSCLC [64]. In bone cancer, solCD44std and v5 are downregulated in all patients regardless of metastasis, limiting diagnostic utility [65]. Similarly, in pediatric sarcoma patients, solCD44 levels are comparable to those in healthy controls, indicating minimal relevance [66].

### 3.2. SolCD44 as a Prognostic Biomarker

Numerous studies have explored the role of solCD44 isoforms as biomarkers of cancer progression, particularly concerning TNM stage, lymph node involvement, tumor size, and distant metastasis. In breast cancer, solCD44v6 levels were significantly elevated in patients with metastatic disease and were positively correlated with the number of metastatic sites [67]. In contrast, solCD44v5 has demonstrated limited prognostic utility because of its increased levels in smokers and individuals with chronic inflammatory conditions [68]. Another study [69] confirmed the prognostic value of solCD44v6, reporting higher mean levels in cancer patients (269.2 ± 94.3 ng/mL) than in controls (179.5 ± 50.7 ng/mL), whereas solCD44std was not significantly correlated with disease status. In HER2-positive breast cancer, elevated serum CD44 levels are associated with worse overall survival, whereas no such association was found in HER2-negative patients [70]. Notably, in triple-negative breast cancer, serum solCD44 levels are higher than those in luminal subtypes (mean: 506.8 ± 175.5 ng/mL vs. 406.4 ± 68.3 ng/mL), suggesting its possible association with more aggressive disease [71].

Prognostic role of solCD44 has also been studied in other forms of cancer. In colorectal cancer, solCD44v6 has consistently been associated with advanced Dukes’ stage, lymph node metastasis, and poor prognosis [72]. Patients with solCD44v6 levels >140.9 ng/mL had significantly worse outcomes than those with lower levels. Subsequent studies confirmed that solCD44v6 remained elevated post-surgery and was positively correlated with tissue expression [73]. Another study [74] revealed that solCD44v6, but not v5, was elevated in patients with bone and liver metastases, with serum concentrations reflecting its expression in primary tumor tissues.

In gastric cancer, solCD44v5 and v6 are elevated in advanced-stage patients and are correlated with tumor invasion, nodal involvement, and metastasis [75]. Both isoforms, although also present in healthy individuals, were significantly reduced following tumor resection, supporting their tumor-associated origin. A larger cohort study [38] further confirmed the prognostic value of solCD44v6 in diffuse-type gastric carcinoma, showing correlations with tumor depth, lymph node metastasis, and clinical stage. However, neither soluble nor tissue CD44v6 was associated with survival in patients with intestinal-type gastric cancer.

In OSCC, serum solCD44 levels increase with histological grade, with the highest levels observed in poorly differentiated tumors [76]. The concentrations in OSCC patients were also significantly higher than those in controls, supporting the value of solCD44 as both a diagnostic and prognostic biomarker. Assessing salivary solCD44v6 in 66 laryngeal squamous cell carcinoma patients revealed significantly higher levels in those with larger primary tumors (T3–T4), supraglottic involvement, nodal metastasis, and advanced-stage disease, suggesting that solCD44v6 has prognostic utility in laryngeal cancer [40]. Strong solCD44v6 expression in recurrent tumor tissue was correlated with poor survival and recurrence, supporting its value as a prognostic biomarker [77]. The study of salivary solCD44v6 in laryngeal carcinoma patients revealed its potential as a prognostic biomarker [78]. Although the evidence regarding the correlation between CD44 isoforms and distinct clinicopathologic subtypes in head and neck cancers remains inconclusive, the findings suggest that solCD44v6 could contribute to subtype differentiation and prognosis in laryngeal cancers. Salivary solCD44 was evaluated as a postsurgical prognostic marker. The mean levels decreased from 70.75 ng/mL pre-surgery to 31.1 ng/mL at six months post-surgery, with no recurrences observed in patients whose levels declined [78].

Another study evaluated the potential of pre-surgical serum CD44 levels as a non-invasive prognostic tool in oral cancer [77]. Serum samples from 64 primary oral cancer patients and 16 healthy individuals were analyzed using ELISA, and solCD44 levels were correlated with clinical outcomes over a median follow-up period of 19.2 months. solCD44 levels were significantly higher in patients compared to healthy individuals and were also elevated in those with local recurrence compared to non-recurrent cases. High serum CD44 levels were associated with reduced survival, while solCD44v6 staining in recurrent tumors was significantly stronger than in primary tumors, correlating with poor prognosis. Patients with both elevated serum CD44 and solCD44v6 expression in tumors showed a strong association with local recurrence and poor survival. These findings suggest that pre-surgical serum CD44 levels, assessed through ELISA, could serve as a reliable, non-invasive method to identify high-risk recurrent tumors and guide post-surgery treatment strategies.

The clinical importance of solCD44 in hematologic cancers has been demonstrated by the detection of solCD44std in patients with acute leukaemias, including acute lymphoid leukemia (ALL) and acute myeloid leukamia (AML), using a custom-made ELISA [79]. Additionally, it was measured in patients with myelodysplastic syndrome or chronic myeloid leukemia (CML) and in healthy individuals. The results revealed that solCD44 was significantly elevated in patients with acute leukemia and CML compared with healthy volunteers. The study of the role of solCD44 in NHL [39] suggested that solCD44v6 concentrations, measured via ELISA, are increased in patients with NHL. This study included 201 patients with Hodgkin’s disease, NHL, or adult T-cell leukamia/lymphoma (ATLL). Patients with aggressive NHL were divided into groups based on the solCD44v6 cutoff of 800 ng/mL. These findings indicated that elevated solCD44v6 in patients with NHL was associated with decreased overall survival and with stage III or IV disease, suggesting that solCD44v6 may be used to predict and evaluate disease progression and survival. Moreover, there was a strong correlation between high serum lactate dehydrogenase (LDH) levels and increased solCD44v6 levels, suggesting that solCD44v6 may be an independent prognostic indicator during cancer therapy in clinical settings and a predictor of disease outcome.

In acute leukemia, solCD44 levels in patients with AML and ALL were approximately four times higher than those in healthy controls before treatment [80]. These levels correlate with leukemic burden and clinical status, suggesting that solCD44 may serve as a prognostic indicator. A minor increase in solCD44 was also noted in bacterial infections. A study was conducted to elucidate the roles of solCD44std and solCD44v6 and their impact on clinical prognosis and outcome [35]. They reported a significant association between increased total solCD44std and solCD44v6 in 90 patients with confirmed B-CLL. The median and mean values of solCD44 were also compared with those of healthy individuals in this study. Moreover, findings indicate a correlation between higher values of both solCD44 variants and progression of disease stage and alteration of lymph nodes. Elevated solCD44std was associated with increased numbers of leukocytes, whereas solCD44v6 was linked to cases of splenomegaly. Taken together, these results provide evidence that both elevated solCD44std and solCD44v6 could play pivotal roles in the pathogenesis of B-CLL and could serve as prognostic parameters in clinical settings to help clinicians assess patients’ conditions and guide treatment.

In ovarian cancer, solCD44v5 and v6 were significantly elevated in patients with advanced FIGO stage (III–IV) disease compared with patients with early-stage (I) disease, whereas solCD44std showed no meaningful correlation with clinicopathological features [32]. Another study analyzed preoperative serum from ovarian cancer patients and reported that solCD44v6 was elevated in those with pelvic lymph node metastases. SolCD44v5 was reduced in progesterone receptor-positive tumors and postmenopausal patients and was associated with shorter relapse-free survival. However, no significant associations with stage or overall survival were found [30].

A study on lung cancer revealed that people with increased serum CD44v6, together with its tissue expression, had more unfavorable outcomes, and serum CD44v6 was an independent prognostic factor in patients with NSCLC. The protein’s standard form, both in tissues and in serum, was not a predictive factor for survival. In addition, soluble protein levels were higher in male patients or smokers than in female participants or nonsmokers who participated in the study [81].

In contrast, limited or no significant correlation was observed in prostate, renal, and pancreatic cancers. In prostate cancer, solCD44v5 levels are lower in cancer patients than in controls, and no correlation was found between solCD44std or v6 and clinical stage [82,83]. In renal cancer, male patients presented lower solCD44 levels than healthy individuals did [84]. A study on pancreatic cancer reported a negative correlation between the solCD44 concentration and clinical prognosis, although data remain scarce [85].

### 3.3. SolCD44 as a Predictive Biomarker

The ability to predict and monitor therapeutic response is critical in cancer management, and numerous studies have investigated solCD44 isoforms, particularly solCD44v6 and solCD44std, for this purpose across various malignancies.

SolCD44v6 has demonstrated predictive value in breast cancer patients undergoing second line hormone- or chemotherapy. Higher concentrations were observed in nonresponders to second-line hormones or chemotherapy (median: 447 ng/mL) than in responders (171 ng/mL), with values ≥ 250 ng/mL in patients with liver metastasis indicating poor therapy response [86]. In vitro studies using breast and endometrial cancer cell lines revealed that hormonal treatments, such as estradiol, tamoxifen, and GnRH analogs, modulated solCD44 levels variably, suggesting hormone-specific regulation of CD44 isoform secretion and a complex relationship with therapeutic response [87].

There is evidence of solCD44 and its isoforms being a predictive biomarker in head and neck cancer. Thus, a study has reported higher pretreatment serum levels of the solCD44 isoforms (std, v5, v6) than in controls, with significant reductions following therapy in head and neck cancer [34]. The median pretreatment values were 327 ng/mL (std), 312 ng/mL (v5), and 211 ng/mL (v6). Although no correlation with clinicopathological variables was observed, pretreatment levels correlated with TNM stage and declined after treatment, suggesting a role in monitoring therapy response. Additionally, tissue CD44 expression is predictive of local recurrence after radiotherapy, prompting further exploration of solCD44 as a noninvasive monitoring tool [88]. In oral and maxillofacial cancers, serum solCD44v6 does not differ significantly across stages but decreases after treatment (surgery, chemotherapy or a combination), suggesting the potential for therapy monitoring rather than diagnosis [89].

While previous studies have shown limited usage of solCD44 in general prostate cancer, a more recent investigation revealed its potential role in identifying docetaxel-resistant castration-resistant prostate cancer [90]. Proteomic analysis of resistant cell lines and patient serum identified elevated CD44 levels as an independent predictor of poor survival. Silencing CD44 restored docetaxel sensitivity, indicating its possible use as both a predictive biomarker and a therapeutic target in treatment-resistant castration-resistant prostate cancer.

Some studies highlighted a link between solCD44 levels and therapy response in gastrointestinal cancer. In patients who underwent surgery for metastatic colon cancer, solCD44 levels significantly decreased three weeks postresection. Elevated solCD44 levels (up to 30.8 nM) were observed in patients with metastatic gastric cancer compared with healthy controls (2.7 nM), with significant reductions following surgery [26]. These findings suggest that solCD44 may reflect the tumor burden and could serve as a noninvasive marker for monitoring postsurgical response. In another study, 14 of 15 patients with metastatic colon cancer presented elevated solCD44 levels, which correlated with disease extent. However, short-term follow-up in chemotherapy-treated patients revealed no consistent association between solCD44 (std and v6) levels and clinical outcome, limiting its reliability compared with established markers such as CEA [91].

Some studies have shown significantly elevated solCD44std in patients with NHL, chronic lymphocytic leukemia (CLL), and acute leukemia, which decreases following successful chemotherapy [49,92]. For example, CLL patients with solCD44std levels above 642 ng/mL had more advanced disease and elevated LDH/β2-microglobulin. However, solCD44v6 was less predictive. While solCD44std has potential as a surrogate endpoint for progression-free survival in CLL patients, defining cutoff values and standardizing it remain challenges. In pediatric leukemia patients, solCD44 levels are significantly higher at diagnosis and decline during remission, confirming its dynamic response to treatment [93].

### 3.4. SolCD44 as a Co-Player with Other Biomarkers

Single biomarkers are often inadequate because they fail to capture the complexity of heterogeneous tumors; therefore, a multiplex assay that detects multiple biomarkers could be advantageous [94] A study by Samy et al. [95] highlighted the clinical implications of serum solCD44 in patients with B-CLL. ELISA assessed serum concentrations of solCD44 and interferon gamma (IFN-γ), which were significantly elevated in patients with B-CLL compared with healthy individuals. This work also demonstrates the prognostic value of CD44 and IFN-γ in disease progression and sheds light on their potential role in the pathogenesis of B-CLL through their suppression and as promising therapeutic approaches. In addition, it is correlated with the level of another B-CLL biomarker (β_2_-microglobulin) and the number of leukemic B cells found in the blood [96]. The protein is also associated with poor prognosis [93]. solCD44 was studied as a metastasis-promoting factor, together with other potential biomarkers (8 in total, including angiogenesis and basement membrane invasion factors), to evaluate its prognostic and therapeutic roles in NSCLC. High levels of CD44 are correlated with increased risk of recurrence and survival probability, together with decreased levels of E-selectin [97]. CD44, transglutaminase 2 (TGM2), and epithelial cell adhesion molecule (EpCAM) were identified as novel plasma markers for endometrial cancer diagnosis using the MAGPIX^®^ System, which employs magnetic nanoparticles coated with antibodies and requires a tiny sample volume (25 µL) [98]. A model using these biomarkers showed high sensitivity (84%) and specificity (100%).

Another set of studies involving multiple biomarkers reported that solCD44 is only a performance enhancer of the assay or has a negative association with cancer. In a case—control study of bladder cancer-associated biomarkers, voided urine samples from 127 subjects, 64 with bladder cancer patients and 63 controls, were examined, and the urine concentrations of four different biomarkers, including the CD44 protein, were assessed via ELISA [94]. The proteins were detected in samples from both the tumor-bearing and control groups. Interestingly, the mean urinary CD44 concentration was higher in subjects without bladder cancer than in those with bladder cancer (117.22 ng/mL vs. 53.09 ng/mL, *p* < 0.0001). Although CD44 was less accurate than CCL18 in the present study, detecting all three biomarkers (CCL18, PAI-1, and CD44) together might improve the model’s performance by reducing error.

A recent study used targeted liquid chromatography–tandem mass spectrometry (LC–MS/MS) (not ELISA, as in almost all other abovementioned studies) to study the role of several biomarkers in colon cancer [99]. CD44, together with three other biomarkers (GC, CRP, and ITIH3), showed the best performance in discriminating regional cancer (lymph node invasion) from localised cancer. However, CD44 was not among the five best biomarkers in the panel with the best performance (70% specificity at over 89% sensitivity).

The urinary concentrations of 14 biomarkers were investigated to determine their diagnostic value for bladder cancer detection [100]. An assessment of voided urine from 127 patients was performed in a case—controlled study in which 64 patients had bladder cancer. Fourteen biomarkers, including CD44, were assessed by ELISA. Thus, the mean and median urinary levels of CD44 are negatively associated with bladder cancer.

### 3.5. Comparison of CD44 Expression in Tissue and Biological Fluid

Several studies have investigated solCD44 and its expression in tissues as a cancer biomarker. A study detected three isoforms of circulatory CD44 (solCD44v8-10) in people with colon cancer [101]. The protein level was lower in those with negative immunostaining results, whereas the amount of CD44v8-10 in the blood was notably increased in patients whose tissues showed a strong response to solCD44v8-10. The average signal (optical density unit) in these individuals was 5.5 times greater than that in cancer patients who did not exhibit solCD44v8-10 tissue reactivity. There is a significant association between the level of serum CD44v8-10 and the presence of solCD44v8-10 in tumor tissues. This study proposes that serum CD44v8-10 levels may serve as a biomarker for detecting the progression of colorectal cancer through the bloodstream. A clinical study was conducted to evaluate CD44 splice variant expression in ovarian cancer using both immunohistochemical and serological analyses [37]. The study involved 22 patients diagnosed with different stages of ovarian cancer, classified according to the International Federation of Gynecology and Obstetrics (FIGO) system, which is used to stage gynecologic cancers based on tumor spread and extent. Researchers have assessed the levels of solCD44std, solCD44v5, and solCD44v6 in both tumor tissue and serum samples. Immunohistochemical analysis revealed minimal expression of solCD44v5 and solCD44v6 in tumor tissues. Similarly, the blood levels of these isoforms did not differ significantly between patients with active disease and those in remission, or between patients with active disease and healthy individuals. The average serum concentrations of solCD44std, solCD44v5, and solCD44v6 were not substantially elevated in preoperative samples. These findings suggest that CD44 splice variants are expressed at low levels in malignant ovarian tumors and that their serum levels do not reliably reflect tumor burden. Additionally, the results of this study indicate that solCD44std levels in serum may be influenced more by hematopoietic activity than by the presence of ovarian cancer. On the other hand, while semiquantitative assessment can distinguish between negative and positive reactions, it lacks the precision needed to evaluate intermediate staining patterns. By using serum for quantitative analysis, it is possible to overcome the limitations associated with semiquantitative immunohistochemical staining.

### 3.6. CD44 as a Component of Serum Exosomes

A new path for studying the role of solCD44 lies in elucidating its function in serum exosomes, which mediate cellular communication. Thus, several studies have isolated the CD44 protein as part of tumor exosomes. Exosomes are a type of extracellular vesicle that originate from endosomes. From a clinical perspective, exosomes are a source of biomarkers [102]. They serve as message carriers in the tumor microenvironment and alter signaling in the cells that receive them [103]. Although exosomes circulate in biological fluids, the CD44 they carry is the membrane-associated form rather than the proteolytically shed soluble CD44, as exosomal CD44 is transferred intact from the cancer cell membrane to recipient cells, similarly to what has been demonstrated in ovarian cancer where exosomal CD44 increases CD44 levels in mesothelial cells and promotes invasion [104]. Exosomes are most commonly isolated and purified via ultracentrifugation or gradient ultracentrifugation. Exosomes containing CD44 were first isolated by differential centrifugation, then lysed and analysed by Western blotting [103].

Interestingly, compared with those derived from nonresistant cells, exosomes derived from chemoresistant cells contained a higher level of CD44. This research suggested that breast cancer cells can disseminate chemoresistance via exosomal proteins, particularly CD44 [105]. Another study identified CD44 in exosomes as a cargo protein that transfers the lymph node metastasis phenotype from cells with high lymphatic metastatic potential to primary gastric cancer cells [106]. As a result, the cells also have increased metastatic potential. When studying how exosomes derived from epithelial ovarian cells mediate metastasis, CD44 was found to be increased on exosomes and to promote invasion [104]. The authors proposed capturing CD44-expressing exosomes as an alternative, less toxic antitumor therapy, similar to HER2osomes.

Investigation of proteins inside extracellular vesicles from glioblastoma patients revealed the upregulation of 6 proteins, including CD44, compared with those in healthy volunteers [107]. Three of these protein panels (including CD44) showed significant increases with tumor progression. Most importantly, the combined detection of five of those biomarkers was able to detect tumor progression. One advantage of studying extracellular vesicles in glioblastoma is that they reflect the actual state of the cells from which they were derived. CD44, together with MMP14 and BCG, is part of a subpopulation of extracellular vesicles for glioblastoma invasion.

### 3.7. SolCD44 in Different Biological Fluids

**Figure 1:**
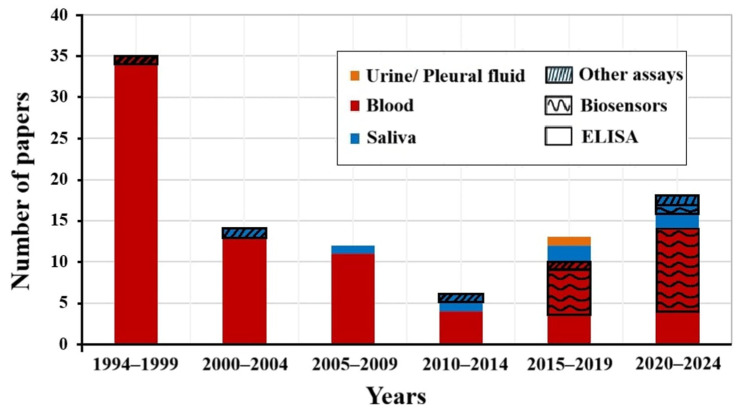
Publications per year for the detection of soluble CD44 protein in biological fluids, based on papers published from 1994–2024.

The soluble form of CD44 has been identified as a cancer biomarker in biological fluids, including serum, saliva, urine, ascitic fluid, and pleural fluid. The analysis of scientific papers published between 1994 and 2024 on the detection of solCD44 protein in biological fluids, broken down by type of fluid (blood, saliva, urine/pleural fluid) and method of analysis (ELISA, biosensors, other analyses), is shown in Figure 1. Most publications were between 1994 and 1999, with almost all using ELISA and focusing on blood samples. Serum is considered the most crucial sample used in diagnostics for three main reasons: it is obtained minimally invasively; it has relatively high stability; and it accurately reflects an organism’s physiological condition [108]. Serum is readily accessible and allows for objective measurement; moreover, the data can be obtained before surgery and may aid in optimal preoperative planning [69]. Serum is a complex medium, and biomarkers are usually present at low levels; efficient methods of detection and characterization are mostly antibody-based assays [109]. However, according to a recent review that mapped sampling types by invasiveness, blood is near the high end of invasiveness [110].

For solCD44, as it is found in biological fluids, urine is the least invasive sampling method, followed by saliva. There is controversial data in the literature on the role of CD44 protein in urine as a biomarker for early diagnosis of bladder cancer. A study [59] showed that urinary CD44 could be used to differentiate an aggressive form of bladder cancer from a low-grade tumor. However, in another study, solCD44 in urine showed no association with bladder cancer [100]; another study showed a negative association with bladder cancer [100]. According to Figure 1, since 2015, biological fluids other than blood (saliva and urine/pleural fluid) have also been studied, possibly indicating a shift toward non-invasive sampling methods.

solCD44 was also found in the saliva of cancer patients. Saliva is produced by the salivary glands to clean and protect the mouth, aid digestion, and help disinfect the mouth. It is considered a significantly diluted body fluid containing electrolytes, nitrogenous products, and proteins [111]. Biomarkers in saliva have been recognized as early indicators of oral and systemic diseases, such as breast, lung, pancreatic, prostate, periodontal diseases, oral cancer, and diabetes mellitus [112], as well as HNSCC, which accounts for most cancers of the mouth, pharynx, and larynx [54]. About 90% of saliva is produced by the salivary glands, which include the parotid gland, submandibular glands, and sublingual glands. Due to its high permeability, biomarkers in the blood can be readily secreted into saliva. Saliva is a potential source of protein biomarkers that could enhance the diagnosis and clinical outcomes of patients with OSCC and breast cancer, using ELISA as the gold standard for biomarker detection. However, this method does not detect many biomarkers and is tedious and expensive [111]. Given that saliva contains hundreds of components that could serve as biomarkers for systemic diseases [112], it is an essential biological fluid for investigating physiological and pathological conditions, such as cancer. It is easily accessible and relatively non-invasive. However, some biological markers are present at low concentrations. Hence, there is a need for improved technologies with lower detection limits [112]. The use of saliva for diagnosis and surveillance is a promising strategy, as the specimen can be collected non-invasively and inexpensively compared to blood or biopsy [113].

Less-represented fluids as sources of solCD44 include pleural fluid and cystic lesions. For instance, a study evaluated the diagnostic potential of soluble adhesion molecules, including CD44, for differentiating benign from malignant ovarian cystic tumors [114]. Serum and cyst fluid samples from 77 patients with various ovarian cystic lesions were analyzed using ELISA kits to measure soluble ICAM-1, solCD44std, and soluble E-cadherin levels. The findings revealed that, while serum levels of sCD44 did not differ significantly between benign and malignant tumors, its concentrations in cyst fluid were notably higher in borderline and malignant tumors than in benign cystadenomas. This suggests that solCD44std, particularly in cyst fluid, may help distinguish malignant from benign tumors, including borderline tumors. Although serum levels of these adhesion molecules showed limited diagnostic value, elevated levels of solCD44std and other markers in cyst fluid highlight their potential as valuable tools to improve the diagnostic accuracy of ovarian cystic tumors.

Other fluids were found not to be a good source of solCD44 for cancer diagnosis. Another study also analyzed levels of solCD44 isoforms in ascitic fluid collected from ovarian cancer patients [36]. They found that the concentrations of solCD44v5 and solCD44v6 were significantly lower in ascitic fluid compared to serum. Specifically, CD44v5 levels were nearly half of those observed in serum, while solCD44v6 levels were also markedly reduced. In contrast, standard CD44 levels showed no significant difference between serum and ascitic fluid. These findings suggest that the tumor microenvironment, as represented by ascitic fluid, may not be the primary source of solCD44v5 and solCD44v6, indicating that these molecules are likely derived from systemic sources, such as immune or stromal cells, rather than directly from tumor cells.

## 4. Assays for CD44 Protein Detection

A wide range of analytical techniques, including ELISA, biosensors, and lateral-flow assays (LFAs), have been used to detect the solCD44 protein. These assays use several types of ligands as biorecognition elements to target the CD44 protein (see Table 2).

### 4.1. Ligands Against solCD44

#### 4.1.1. Hyaluronic Acid

The primary ligand of CD44 is hyaluronic acid (HA); the binding of CD44 to HA contributes to a wide variety of physiological and pathological processes. For example, glioblastoma multiforme, one of the most malignant brain tumors, expresses high levels of the HA receptor CD44 [18]. CD44, the primary cell adhesion receptor expressed in cancer stem cells (CSC), has a specific extracellular domain in the N-terminal region that binds HA in the ECM; this domain is called the HA-binding domain, as shown in the Graphical abstract. CD44–HA interactions activate cellular signaling pathways that promote cancer cell proliferation, invasion, and metastasis [138]. HA-CD44 affinity is primarily used in targeted cell imaging and drug delivery. The ability of HA to bind CD44 rapidly and effectively is vital for extracting CD44 from complex media, such as serum [109]. HA has been used as a ligand in several biosensors for detecting soluble proteins [115,116,117] and in nanoparticle-based assays [109].

#### 4.1.2. Antibodies

Antibodies are widely used as ligands in commercial and home-based ELISAs. In a multitude of biosensors, as shown in Table 2, there are now more than 140 commercially available antibodies against CD44. Despite the large number of available antibodies against CD44, most are the same original monoclonal antibodies or target similar regions of the protein. Given the distinct functions of CD44 isoforms and the sequence variations in both antibodies and antigens, the number of available monoclonal antibodies is considered insufficient, and efforts to develop new antibodies are ongoing. For example, researchers have obtained four new monoclonal anti-CD44 antibodies derived from mouse B cells injected with a plasmid expressing the CD44 isoform 12 [139]. Based on these results, the authors concluded that the four monoclonal antibodies (mAbs) targeting the terminal, extracellular, conserved domain of CD44 bind to these peptides only after deglycosylation.

#### 4.1.3. Aptamers

Aptamers are another class of ligands used for solCD44 detection. Aptamers are single-stranded synthetic DNA or RNA oligonucleotides that can bind to desired cell surface molecules with high affinity and specificity through structure recognition; they are selected in vitro from a synthetic nucleic acid library via the SELEX (systematic evolution of ligands by exponential enrichment) method [42]. These oligonucleotides have dissociation constants in the pico- to nanomolar range and can be combined into a stable, unique three-dimensional structure for specific coupling to target molecules. The advantages of aptamers are their high chemical stability, including heat resistance, ease of synthesis, and flexibility of modification [132].

Aptamers often achieve binding affinities comparable to monoclonal antibodies. For instance, CD44-targeting aptamers, such as CD44-Apt1, exhibit nanomolar affinity in the range of 1.22–2.09 nM and remain intact in 90% human serum for up to 96 h, demonstrating their stability [42]. Unlike antibodies, which require strict storage conditions, and are costly and time-consuming to produce, aptamers can be synthesized at low cost, exhibit high chemical and thermal stability, and flexible chemical modification. Furthermore, the CD44 aptamer demonstrated good analytical performance, enabling the detection of soluble CD44 across a broad linear range of 0.1–1000 ng/mL with the limit of detection LOD of 0.087 ng/mL, together with strong specificity against common serum interferents and good stability during 14 days of storage at 4 °C [137].

A DNA aptamer targeting the recombinant human HA-binding domain of CD44 has been selected by optimizing thio-thio substitutions within the aptamer sequence [128]. The results revealed that selected thioaptamers (monothiophosphate-modified) aptamers bound to CD44-positive human ovarian cancer cell lines but failed to bind to the CD44-negative cell line. The thioaptamers showed significantly stronger, high-affinity specific binding to the HA-binding domain of CD44 (a highly conserved sequence across other CD44 splice variants). They could not bind another HA-binding protein with 32.3% sequence identity to CD44. One of these thioaptamers was then used in another study to construct a biosensor for measuring solCD44 in serum samples [137].

All other aptamers targeting CD44 were originally selected against CD44-expressing cells (not solCD44) during SELEX and showed affinity for the extracellular domain of CD44. The binding of some of the aptamers to solCD44 was also confirmed experimentally, as in previous work [42]. Aptamers targeting two isoforms, solCD44v1 and solCD44v8 10, were selected from live hepatocellular carcinoma (HCC) cells via the loss-of-gain cell-based SELEX method and next-generation sequencing. The study results revealed high affinity and sensitivity of CD44-Apt1 for both proteins.

Aptamers targeting various forms of CD44 are also available. DNA aptamers that specifically bind to CD44 exon v10 were selected via SELEX to inhibit breast cancer cell migration [140]. The inhibition of tumor migration by aptamers targeting exon v10 of CD44 has been confirmed. The use of such aptamers could also be necessary for cancer diagnosis, as the standard form of the protein can be distinguished from its isoforms, given that most ELISA kits are not isoform-specific. While most aptamers targeting CD44 are DNA-based, some RNA-based aptamers, such as those isolated by SELEX from a 2′-fluoropyrimidine-modified RNA library, are available [129].

#### 4.1.4. Emerging Ligands Against solCD44

The large size of mAbs (four polypeptide chains, 150 kDa) hinders their ability to access tumor cells in vivo. Nanobodies (15 kDa) and nanobody-based human heavy chain antibodies (75 kDa) can overcome these obstacles due to their small size, high stability, high solubility, excellent in vivo tissue penetration, and ability to be chemically conjugated to specific sites on drugs, other nanobodies, peptides, etc. [141]. There are currently few studies on CD44 nanobodies. Nanobodies recognizing CD44-expressing cells were successfully isolated using phage display, particularly the cell-panning technique [142]. The development of signal peptides that can direct cytoplasmic proteins from the cell to the media is an essential step in the production of the recombinant CD44 protein. Based on an in silico investigation of suitable signal peptides for the recombinant production of CD44 nanobodies, five peptides (CSGA, TRBC, YTFQ, NIKA, and DGAL) were selected as suitable candidates [143].

Peptides are also attractive ligands compared with antibodies because they are selected in vitro, are more stable, have a lower molecular weight, and are less toxic [135]. Peptides are usually selected using phage display. It is a high-throughput method for selecting particular ligands from a peptide library [48]. Many other peptides have been chosen not as ligands in assays but rather to modulate CD44 activity and study its effect on cancer development [133,135,144,145].

Another emerging class of ligands for CD44 detection is molecularly imprinted polymers (MIPs). MIPs are synthesized by polymerising monomers in the presence of a template molecule using the molecular imprinting method [146]. MIPs are stable polymers with molecular recognition sites, have an affinity for the selected “template” molecule, and can specifically rebind the target molecule. The advantages of MIPs include their high selectivity, high capacity for recognizing and capturing target molecules, stability, and resistance to a wide range of environmental pH and temperature conditions. The functional abilities of MIPs correspond to the interactions of natural receptors, enabling selective retention of the target molecule, as in an antibody–antigen interaction. MIPs with CD44 as the template protein were developed using alginate gel as the functional monomer [132]. The MIPs were shown to be very specific to the target molecule.

### 4.2. Commercially Available Assays

At least two assay types are commercially available for detecting solCD44. ELISA is based on a sandwich-based assay that involves two antibodies: capture antibodies coated on the microtiter plate to capture the protein of interest and a secondary antibody conjugated with enzymes (usually horseradish peroxidase, alkaline phosphatase or β-d-galactosidase) that will digest its substrate to produce a chromogenic species that is detected via a plate reader or the naked eye [147]. An ELISA kit from Bender MedSystems, for example, uses two particular murine antibodies against solCD44 [41]. Most of these ELISA kits (if not all) allow quantitative detection of all circulating CD44 isoforms, including the standard protein and its variants. The majority of the studies discussed in this paper quantified solCD44 by ELISA.

**Figure 2:**
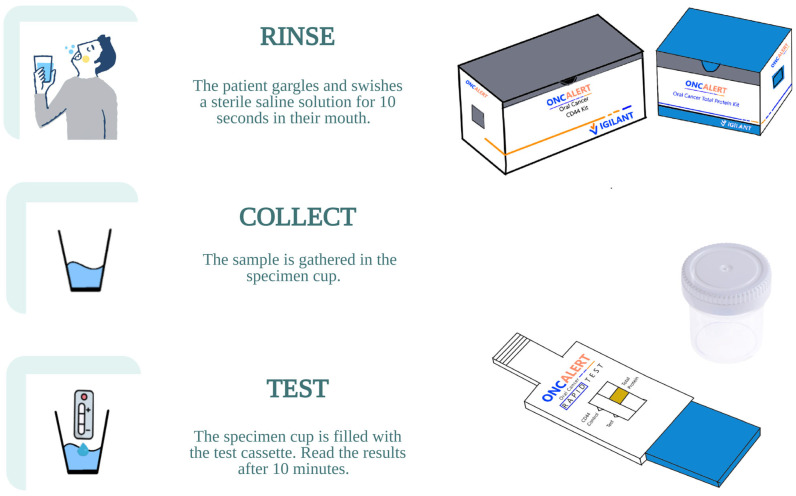
Schematic representation of the detection of the CD44 protein in saliva via the OncAlert™ kit.

The OncAlert™ rapid test kit is another example of a commercially available product sold to detect solCD44 and total protein (Figure 2). Compared with ELISA, it uses saliva as the sample for analysis and employs a lateral flow mechanism, similar to that of a pregnancy test, providing rapid results for oral cancer screening. This device consists of a plastic-molded tube with two test strips, one coated with CD44 and the other with total protein, and a 5 mL saline solution for oral rinsing and gargling. The results were obtained 10 min after the kit was inserted into the collection cup containing the sample. The quantity of CD44 present in the sample is indicated by a visual line proportional to the concentration of human CD44. Figure 2 below illustrates the three-step procedure for using the OncAlert™ test kit.

### 4.3. Biosensors and Other Assays

As shown in Figure 1, although the total number of articles decreased after 1999, methodological diversity gradually increased, especially with the advent of biosensors and other analytical methods starting in 2015. From 2020 to 2024, there has been a marked increase in biosensor-based research, especially the use of blood and saliva, indicating a growing interest in advanced detection technologies. Biosensors for detecting solCD44 can be classified into three categories: electrochemical, photoelectrochemical, and optical fibre-based. Among electrochemical biosensors for solCD44, electrochemical impedance biosensors, which measure impedance via a three-electrode system with an electrochemical analyzer as the protein concentration changes, are more common. One such biosensor uses aptamers as ligands to detect proteins in serum samples [137]. The binding of the target to the aptamer alters its structure, thereby enhancing the flow of electroactive species toward the working electrode and decreasing impedance. Among biosensors that exhibit ultrasensitive, wide-concentration-range detection are a sandwich-type electrochemical biosensor using an enzyme-free signal amplification strategy [133] and a spherical ball resonator-based optical fibre biosensor with a simple fabrication method [121].

Since specific isoforms of CD44 may play a more critical role in certain cancers, their quantitative detection is crucial. One of these methods involves developing isoform-specific ELISAs [43]. Specifically, the lack of tests for CD44v3 was addressed by creating a sandwich ELISA, which was shown to be sensitive, accurate, and reliable for detecting this CD44 variant [119]. In one study, ion exchange, immunoaffinity chromatography, and Western blot analysis were used as alternative methods to isoform-specific ELISA to examine the roles of different CD44 isoforms in colon cancer [43].

Biosensors fabricated for exosomal CD44 detection are also available. In one study, two types of label-free biosensors based on titanium nitride nanoholes were developed: one using an atomic force microscope and the other using localized surface plasmon resonance [148]. Exosomes are nanoscale vesicles (30–100 nm) with an endocytic origin that function to transport proteins, mRNAs, or other molecules between cells [104]. LOD of exosomal CD44 for the biosensors were 5.29 × 10^−1^ μg/mL and 3.46 × 10^−3^ μg/mL in terms of exosome concentration. Although the biosensors evaluated exosomes from malignant mouse samples, they offer an interesting outlook for using exosomes as analyzable samples.

Several strategies have been developed to optimize the detection of soluble proteins, including simplifying biosensor development (e.g., easier fabrication and simplified functionalization) and improving biosensor properties, such as antifouling and long-term stability. One-step surface functionalization was used to construct a photoelectrochemical biosensor for the detection of solCD44, which features a hybrid antifouling surface [116]. Another electrochemical biosensor developed for CD44 protein detection demonstrated long-term stability and was successfully used to detect CD44-expressing cells [115]. A fluorescence resonance energy transfer assay resulting from the interaction of fluoresceinamine-HA with a cationic conjugated polymer was developed [149]. This method provides a fast, cost-effective, and visual means of detecting circulatory CD44. Soomro et al. [117] developed a hybrid photoelectrochemical platform that was demonstrated to detect proteins in real blood serum due to the antifouling surface of the biosensor. An optical biosensor based on a low-cost, easy-to-fabricate spherical fiber-optic tip sensor was developed to quantify the CD44 protein [128], with follow-up work achieving an ultralow LOD over a wide concentration range and in vitro studies that mimic blood flow [121].

A dual electrochemical biosensor that combines the natural ligand of CD44 (HA) with ultra highly specific MIPs into a single flexible electrode for detecting the CD44 biomarker was developed [132]. In the developed dual-channel screen-printed electrodes (SPEs), the synthesized MIPs against CD44 were immobilized on one channel, while the second channel was loaded with natural HA probes. The advantages of this development include excellent specificity, antifouling properties, and biocompatibility of MIPs and HA; satisfactory stability of SPEs; and high sensitivity of electrochemical methods. This electrochemical biosensor platform can serve as a valuable tool for diagnosing human cancer by recognizing CD44-overexpressing cell surfaces, such as those of breast cancer cells. Combining biosensors with other assays is also common. Magnetic nanoparticles functionalized with HA were first used to extract and detect the protein from serum; more importantly, the extracted proteins were subsequently analyzed via mass spectrometry [109]. Dot blot analysis is another form of assay which was used for solCD44 detection in some of the studies [92,150,151].

The CD44 protein not only has several isoforms but also has several posttranslational modifications (N- and P-linked glycosylation, phosphorylation, sulfation, and domain cleavage) [8]. Therefore, some studies have focused on detecting post-translationally modified forms of CD44. CD44 glycosylation affects its binding to many important ligands (HA, fibronectin, collagen, etc.) and thereby regulates the tumor microenvironment and cancer cell fate [152]. A solid-phase proximity ligation assay for detecting the glycosylated form of CD44 was developed. As a result, the method outperformed ELISA in terms of dynamic range and sensitivity [153]. However, the technique is not label-free and can be labor-intensive because it requires an additional step during real-time PCR.

### 4.4. Performance of the Assays

**Figure 3:**
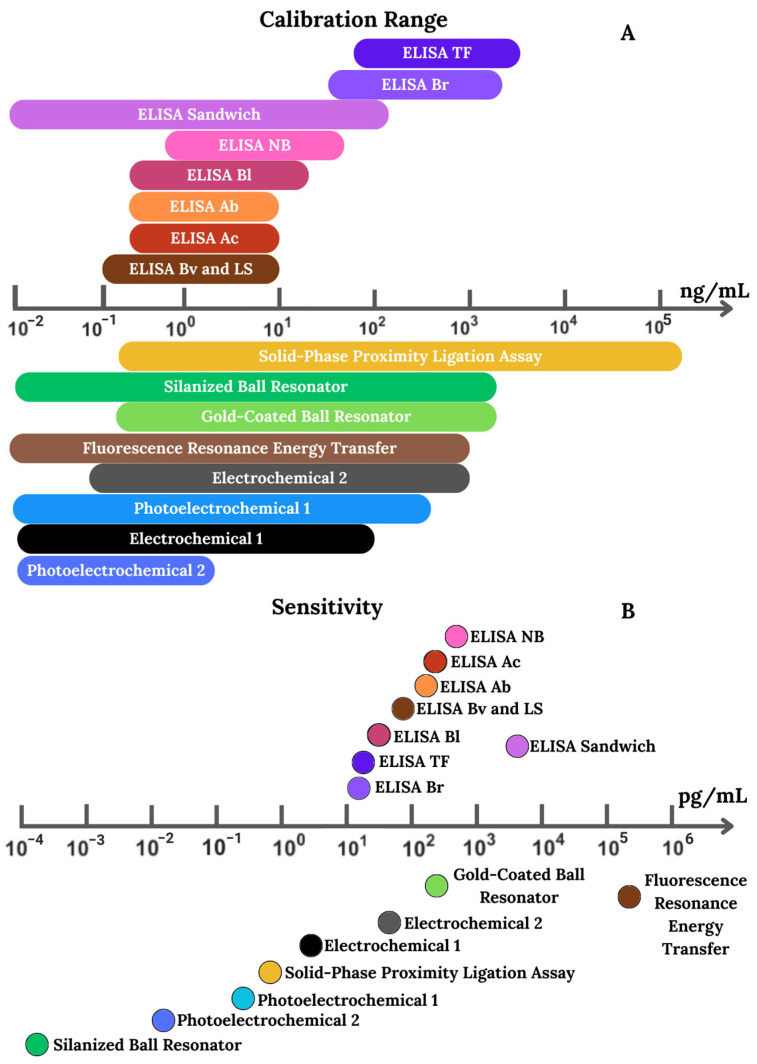
Calibration (**A**) and sensitivity ranges (**B**) of commercially available ELISA kits, biosensors, and other assays for the detection of the CD44 protein in various biological fluids. Commercial ELISA kits: NB—Novus Biological, Ac—Abcam, Ab—Abnova, TF—Thermo Fisher Scientific, BR—Bio-Rad, Bl—BioLegend, LS—LifeSpanBioSciences.

A brief overview of the main detection platforms is provided to contextualize their analytical performance and highlight differences in sensitivity across methods. This helps clarify how commercial assays compare with more advanced sensor-based technologies in terms of applicability for solCD44 detection. Figure 3A shows the calibration and sensitivity ranges of available kits for detecting the CD44 protein in various biological fluids. These kits were designed to detect CD44 protein (standard and isoforms 5, 6, and 9) in biological fluids such as serum, plasma, tissue homogenates, and culture supernatants, and some kits can also be used for urine and amniotic fluid. Figure 3B compares the sensitivity (pg/mL) of commercial ELISA kits and sensor-based methods for CD44 detection. Among ELISAs, kits from Novus Biologicals (NB), Abcam (Ac), and Abnova (Ab) perform best, with detection limits close to 0.01–0.1 ng/mL—although Thermo Fisher and Bio-Rad outperform them slightly (0.016 and 0.0156 ng/mL, respectively). FRET-based immunoassays lag behind other methods, with a sensitivity of approximately 170 ng/mL, making them less suitable for CD44 detection. Sensor-based methods, however, surpass ELISA methods. Photoelectrochemical platforms reached 0.014 pg/mL, and solid-phase proximity ligation assays reached 715 fM. The silanized ball resonator stands out for its sensitivity of 107 ag/mL; however, it requires complex and costly instrumentation. While ELISA kits are more accessible and widely used, sensor-based methods offer higher LODs for the detection of ultralow-level biomarkers.

## 5. CD44 Biomarker: Cell vs. Soluble Protein

Several studies have compared CD44 in tissue with its soluble form. The results of immunohistochemistry (IHC) for CD44 in tumor biopsy samples and ELISA for CD44 protein in serum correlated with those of several studies [73,76,101,154,155]. A study detected three isoforms of circulatory CD44 (solCD44v8-10) in people with colon cancer [94]. The protein level was lower in those with negative immunostaining results, whereas the amount of CD44v8-10 in the blood was notably increased in patients whose tissues showed a strong response to solCD44v8-10. The average signal (optical density unit) in these individuals was 5.5 times greater than that in cancer patients who did not exhibit solCD44v8-10 tissue reactivity. There was a significant association between the level of serum CD44v8-10 and the presence of solCD44v8-10 in tumor tissues. This study proposes that serum CD44v8-10 levels may serve as a biomarker for detecting the progression of colorectal cancer through the bloodstream.

Evaluation of the expression of solCD44std, solCD44v5, and solCD44v6 in ovarian cancer through both IHC and serological analyses suggested that CD44 splice variants are expressed at low levels in malignant ovarian tumors and that their serum levels do not reliably reflect the tumor burden [37]. Additionally, the results of this study indicate that solCD44std levels in serum may be influenced more by hematopoietic activity than by the presence of ovarian cancer. A systematic review and meta-analysis on the role of CD44 in colorectal cancer suggested that IHC is a more reliable method than ELISA, since serum CD44 levels are affected not only by cancer but also by immune system activity [156].

There are several reasons why plasma protein can be used as a liquid biopsy: an increased understanding of protein complement of cell types, current improvements in protein assay technology, and the possibility of early cancer detection with life- and cost-saving effects [3]. To cover different stages of disease development, it is essential to have proteins that respond to the following characteristics: accessibility, sufficiency, and reliability [157]. Soluble proteins in serum or plasma can serve as a cheap, minimally invasive tool for diagnosis, including early diagnosis, risk stratification, treatment adjustment, disease prognosis, and disease progression monitoring [158]. Cancer cells/tissues secrete various proteins, including enzymes, cytokines, and growth factors, which play distinct roles in physiological processes. Partially, some secreted secretome can be measured in the bloodstream, potentially serving as biomarkers with easier access than tissue proteins [158].

Table 3 presents a comparative analysis of the cell-surface-expressed and soluble forms of CD44 as cancer biomarkers. Disseminated cancer cells are a good indicator of tumor burden and metastatic risk. Detailed molecular properties of tumours can be derived from studies of these cells. They carry essential information about the tissue, such as DNA, RNA, and other molecules [3]. The circulating CSC population from colon cancer patients has a distinct set of markers, including CD44 [159]. These cells formed spheroids, indicating the presence of tumor-initiating cells. In gastric cancer patients, CTCs expressing CD44 and EpCAM, sorted by flow cytometry, are highly enriched compared with healthy individuals. The number of these cells is associated with tumor progression and venous invasion. The number of these patients also decreased after surgery or chemotherapy [160]. IHC is the most widely used method for analyzing CD44 in tissues. The staining intensity is scored semi-quantitatively [161].

## 6. Conclusions and Future Perspective

**Figure 4:**
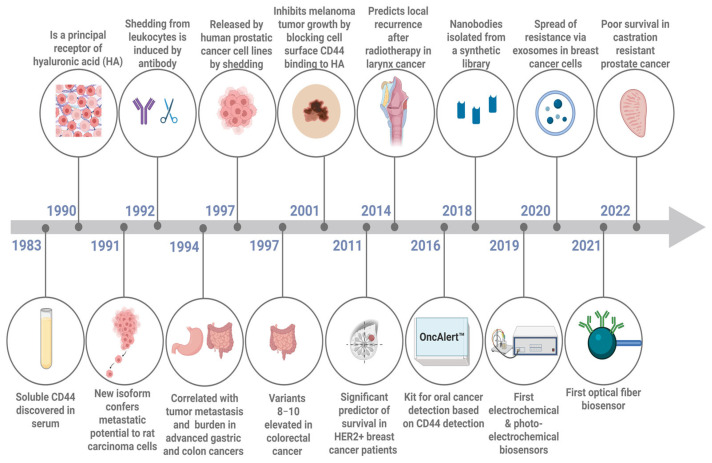
A timeline of the most essential milestones in soluble CD44 for cancer diagnosis. Created in BioRender. Bekmurzayeva, A. (2025) https://BioRender.com/plioj9x.

Our understanding of the role of solCD44 in cancer has evolved significantly, starting with a surge of work evaluating its role as a cancer biomarker in the 1990s, followed by somewhat reduced interest in the 2000s. Subsequent discoveries highlighted its diverse roles, leading to a reevaluation of its significance across various cancer types. Based on the studies discussed, a map of key advancements in solCD44 research from 1983 to 2022 is shown in Figure 4. In 1983, solCD44 was first identified in serum, followed by its identification as a primary receptor for HA in 1990, marking foundational breakthroughs. Between 1991 and 1994, solCD44 was linked to cancer metastasis, and in 1992 and 1997, its shedding from leukocytes and cancer cells was documented. In 2001, the role of solCD44 in inhibiting melanoma progression was recognized. In 2011, solCD44 was associated with predicting laryngeal cancer recurrence and was identified as being overexpressed in HER2-positive breast cancer. By 2022, it was implicated in resistance mechanisms in advanced prostate cancer. Technological advancements include the development of diagnostic tools for oral cancer in 2016, the first electrochemical and photoelectrochemical biosensors in 2019, and the introduction of the first optical fiber biosensor for solCD44 detection in 2021. These discoveries highlight the critical role of solCD44 in cancer research and its potential for clinical applications.

**Figure 5:**
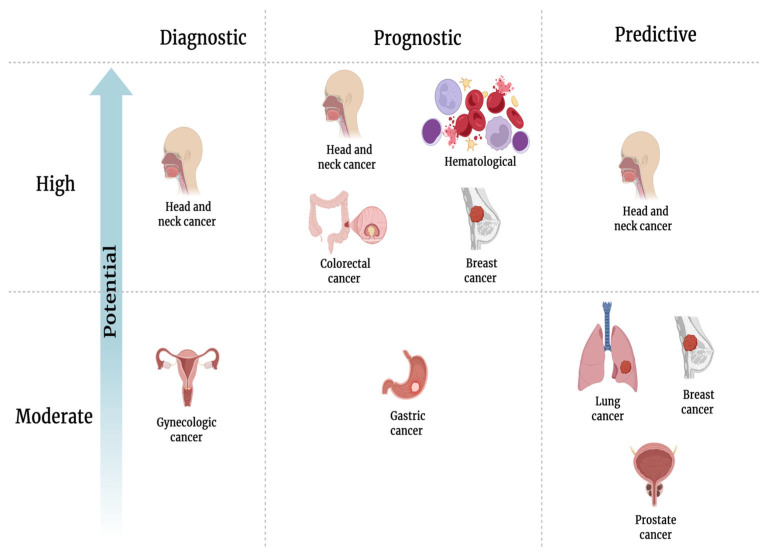
Diagnostic, prognostic and predictive value of soluble CD44 protein in biological fluids in different types of cancer based on the studies discussed in the paper: a rough estimation. Created in BioRender. Nurlankyzy, M. (2025) https://BioRender.com/djj19sj.

SolCD44 shows strong diagnostic potential in HNSCC, such as oral cancer, while serving as a valuable prognostic and predictive biomarker in others, as supported by several studies as shown in Figure 5. According to the diagnostic, prognostic and predictive potential of solCD44, different types of cancer could roughly be divided into two categories: (1) ‘high”—if there are at least 3 strong supporting studies; (2) “moderate”—if there are at least 2 supporting studies, and 2 or 1 of the studies are old ones (published earlier than 2000) and are not supported by recent studies. Considering the fact that cancer types are further divided into subtypes, clinicopathological factors considered vary among studies, and different solCD44 isoforms were detected. The framework of Figure 5 is intentionally simplified and is meant only to provide a broad, approximate overview of our current understanding of the role of solCD44 in cancer diagnosis. Details of some of the studies on which Figure 5 was based are shown in Table S1.

Regarding the early diagnosis of cancer, salivary solCD44, especially solCD44v6, is promising for the noninvasive detection of oral, head and neck, and laryngeal cancers. Rapid tests such as OncAlert™ facilitate point-of-care applicability. HNSCC is a severe and life-threatening condition, primarily due to its frequent diagnosis at an advanced stage. While current cure rates are around 50%, early detection has the potential to raise them to over 80% [54]. Conversely, serum solCD44 appears better suited for prognostic monitoring than for early diagnosis, as its levels often correlate with recurrence and poorer survival outcomes than those of early disease stages. Overall, solCD44v6 stands out as a consistent and reliable isoform associated with TNM staging and metastatic potential across multiple cancer types, particularly colorectal, gastric, breast, oral, and ovarian cancers. Its expression in both serum and tumor tissue, and its persistence after surgery, support its importance as a tumor-derived biomarker with promising applications.

Distinguishing cancer subtypes is essential for guiding appropriate therapy, especially in the era of precision medicine. SolCD44 isoforms, particularly solCD44v6 and solCD44v5, show potential for differentiating aggressive subtypes and identifying therapy-resistant forms in cancers such as breast, prostate, bladder, and laryngeal carcinoma. The expression patterns of these genes may provide valuable insights into tumor behavior and support individualized treatment strategies. Further research is needed to validate their clinical utility and establish standardized thresholds for subtype classification. Overall, solCD44 isoforms, particularly solCD44std and solCD44v6, show promise as biomarkers of therapeutic response in several cancers. Their levels tend to decrease following effective treatment and correlate with tumor burden and progression. However, inconsistent findings, limited specificity, and variability across cancer types underscore the need for standardized assays and larger, longitudinal studies to validate the clinical utility of solCD44 for therapeutic monitoring.

The clinical utility of CD44 depends on the cancer type and the form being studied. For some cancers, tissue expression analysis is more informative, whereas in others, such as oral cancer, the soluble form of CD44 may provide greater diagnostic accuracy. Recent advances in exosome research have revealed the role of CD44 in the spread of chemoresistance, further highlighting its clinical importance. While serum sampling is convenient, cellular and tissue-based studies often yield more profound insights into CD44 function. Consequently, the application of CD44 and its variants should be tailored to specific cancer types to maximize their diagnostic and prognostic potential. Continued research is essential to refine its applications and fully integrate CD44 into personalized cancer care. Every biological fluid containing solCD44 has its own benefits and analytical difficulties. The best choice of fluid depends, first of all on the specific clinical situation and testing needs. While invasive fluids like blood typically provide more accurate results and have more existing research data for comparison, obtaining them often is not practical for repeated testing. On the other hand, fluids collected non-invasively, such as saliva and urine, although are easier to obtain in practice, their use requires strict procedures for consistency and thorough testing to confirm accuracy. Method of collection of biological fluids, especially saliva, may have an effect on the obtained results. Thus, the oral rinse procedure could yield significantly different levels of the tested biomarkers compared to two other collection methods (unstimulated and chew-stimulated saliva).

Based on the works discussed above, the identified challenges in using the solCD44 protein as a cancer biomarker are outlined below. Solid tumors are heterogeneous and therefore composed of cancer cells that vary by subtype; this results in differences in clinical and pathological manifestations, including reported differences in the role of CD44 across breast cancer subtypes and specific patient cohorts [70]. Actual cutoff value for the solCD44 protein in biological fluids have been determined for a very limited number of cancers, such as NHL and cervical cancer [1]. Variations in the effects of increased CD44 expression and in the prognosis of patients with the same cancer type may be primarily due to differences in methodology [189]. Another factor affecting the results could be the time between the protein measurement and the sample collection date, as prolonged or improper storage conditions can decrease protein levels. Concomitant illnesses or habits, such as smoking, were not considered in many of the studies. An earlier study (1997) reported elevated levels of isoforms 5 and 6 in normal individuals who were smokers [190]. Serum levels of the CD44 protein were also investigated in several noncancerous conditions. Its levels differ from normal in several noncancerous diseases: decreased in chronic pancreatitis [191], increased in systemic sclerosis [192], elevated in sarcoidosis uveitis [193], elevated in smokers [162,190], and increased in acute renal rejection [163], hemodialysis-dependent renal failure, and chronic inflammatory bowel disease [91].

As single biomarkers often fail to capture the complexity of heterogeneous tumors, multiplex assays that detect panels of biomarkers may provide a more effective diagnostic and prognostic solution. Exemplary biomarkers that can be used in concert with CD44 include IFN-γ (and β2-microglobulin) for B-CLL cancer, E-selectin for NSCLC, and TGM2 and EpCAM for endometrial cancer. CD44 should be seen as a team player rather than an independent marker, working in concert with other biomarkers and clinicopathological parameters. Combining CD44 with other markers may ultimately be the key to advancing personalized medicine and improving patient outcomes. Among the emerging and intriguing studies are exosome research findings showing that the CD44 protein spreads metastatic potential and chemoresistance.

Future research on CD44 should prioritize distinguishing its isoforms to understand better their specific clinical roles and significance. Compared with traditional approaches such as ELISA, advanced detection methods, such as biosensors, could offer greater accuracy and efficiency in clinical trials. Before biosensor methods can be included in formal directives and inform regulatory choices, they need stringent validation to prove they are either as good as or better than current standard methods in terms of analytical performance. Many of the reported studies on biosensing of solCD44 indicate that they perform exceptionally well in controlled laboratory settings; however, their effectiveness could be decreases when exposed to real complex fluids (serum, urine, saliva). This drop in performance is partially caused by interference from the fluid’s composition (“matrix effects”), unintended binding of molecules (nonspecific binding), and other biochemical interferences. Another issues largely stem from the ligand when it does not completely guarantee selectivity causing cross reactivity. In order to ensure high selectivity of the biosensor and given a lower concentration of target protein (solCD44) compared to interfering substances such as albumin which are present in a much higher concentration, level of cross-reactivity must therefore be exceptionally low [164].

Very few biosensor designs developed in laboratories have become commercially available; this also applies to solCD44 biosensors. The limited transition of biosensors to the market can be attributed to the absence of standardized methods, inadequate testing, and insufficient measurement practices for the materials used to create these sensors [165]. One way to overcome these obstacles is by adopting standards similar to the “standards for reporting optical biosensor experiments” (STROBE) [166], standard procedure detailing the crucial information that must be reported when describing optical biosensor tests, allowing reviewers and readers to fully comprehend and duplicate the experimental setup. Another way to improve this is by adopting standards for pre-analytical and analytical validation according to Blood Profiling Atlas in Cancer (BLOODPAC) [167] in order to enhance patient care and outcomes and better inform medical decisions. BLOODPAC is a collaborator-funded organization whose goal is to expedite the development, validation, and clinical application of liquid biopsy diagnostics (specifically those that utilize ctDNA). Pre-analytical phase includes instructions detailing the proper process for blood sample collection, handling, and storage. Analytical validation part covers vital characteristics of the assays such as sensitivity, specificity, and reproducibility. Establishing these strict standards could make it easier for test assay developers and governing bodies to assess the performance of the assays uniformly, guaranteeing they achieve high quality necessary for use in patient care.

## Figures and Tables

**Table 1:** Different names have been used for the soluble CD44 protein in the literature; those used in this work are listed below. References [1,17,30,31,32,33,34,35,36,37,38,39,40,41,42,43,44,45].

Name of the Protein in the Current Work	Description	Name of the Protein in Other Works	Ref.
solCD44std	- Standard form of CD44 (wild type) - Non variant	- standard CD44 - CD44std - CD44standard - sCD44std - CD44s - sCD44-st - sCD44st - sCD44s	[30,31,32,33,34,35]
solCD44v1-v10	- Soluble variants (isoforms) of the CD44 protein, which arise due to alternative splicing of variable exons in the mRNA of CD44; - v1 (variant 1) refers to isoform 1	- CD44v1 - sCD44v5 (sCD44-v5) - sCD44var v6 - isoform 1 - CD44E (CD44v8-10)	[30,36,37,38,39,40,41,42]
solCD44	One of the following: - general idea of soluble CD44 protein in serum (circulatory), saliva, urine, or other biological fluids - Total soluble CD44 (standard and isoforms); - When protein type (standard or isoform) is not specified in the works: the standard form or variant form is not shown.	- sCD44 - solCD44 - SolCD44 - circulating CD44 - soluble CD44	[1,18,36,43,44,45]

**Table 2:** Different diagnostic probes (ligands) targeting extracellular/soluble CD44 and their use in assays. References [45,115,116,117,118,119,120,121,122,123,124,125,126,127,128,129,130,131,132,133,134,135,136].

Type of Probe/Ligand	Advantages	Examples of Ligands	Use in Assays	
			Soluble	on Cells or Tissues
HA	- Natural ligand - Also used as an anti-fouling agent	(C_14_H_21_NO_11_)_n_	- Biosensors [115,116,117]	- Colorimetric assay using nanoparticles [118]
Antibodies	- A well-known ligand - Commercially available - Some were shown to inhibit oncogenesis	- More than 140 are known - P4G9, P3D2, P3A7, P3G4 - C44Mab−46 (IgG1, kappa)	- All commercial ELISA kits - Customized ELISA [119] - Optical fiber biosensors [120,121,122,123,124] -Electrochemical [125,126] - OncAlert™ kit [45]	- Western blot analysis - FACS - IHC
Nanobodies	- Small size - High stability - High solubility - Excellent tissue penetration in vivo - Chemical conjugation at a specific site (e.g., to drugs)	- 11C12	- NF	- Nanoparticle-based assay [127]
Nucleic acid aptamers	- High chemical stability - Flexibility of modification - Selected in vitro - Ease of synthesis	**-** TA1 [128]—thioaptamer that binds the HA binding domain - Apt1 [129] - s5 [130]—binds the HA binding domain - AS1411 [131] - Dual-responsive aptamer [132]	- Electrochemical biosensors [137]	- Sensing platform for cells [119] - FACS [129] - Fluorescent microscopy [129]
Peptides	- Selected in vitro - Stability - Low molecular weight	**-** A5G27 [133] - P3 and P6 [134] - PDL-P7, P6 (dual peptide) [135]	- NF	- Polymeric target recognition system [135] - IHC [48,135] - FACS [135] - Electro-chemical biosensors [136]
Molecularly imprinted polymers	- Great selectivity - High capacity for recognition and capture (re-binding) - Stability (pH and temperature)	- Protein MIP [132]	- Electrochemical biosensor [132]	- NF

ELISA—enzyme-linked immunosorbent assay; FACS—fluorescent activated cell sorting; IHC—Immunohistochemistry; NF—not found.

**Table 3:** Comparative analysis of tissue-based and soluble forms of CD44 as a cancer biomarker. References [161,162,163,164,165,166,167,168,169,170,171,172,173,174,175,176,177,178,179,180,181,182,183,184,185,186,187,188].

Criteria	CD44 Expressing Cells/Tissues	Soluble CD44 Protein
Availability	Tissue Serum (circulating CSC)	Serum Saliva Urine Ascitic, pleural fluid
Systemic reviews and meta-analysis	28	Not found
Available assays	Immunohistochemisty Microfluidic platforms Flow cytometry MNP-based assays Electrochemical biosensor QCM biosensor Optical fiber-based biosensor	ELISA OncAlert™ Western blot Electrochemical biosensor Optical fiber-based biosensor
Commercially available assays	MNP-based assays	ELISA OncAlert™
Main strengths	Independent of shedding Further processing of cells for additional information More detailed molecular characteristics of cancer A more direct measure of tumor load and risk of metastasis	Present in different biological fluids which are more available than tissue Use in screening of large population is possible Improvement of performance of tissue-based assays
Main drawbacks	Need processing: tissue biopsy, enrichment from other types of cells More sophisticated assays to detect Not all CSC might express CD44 (CD44^low^ or CD44^−^)	Many isoforms available Levels might be affected by immunological response Elevated/lowered in some non-cancerous conditions (including smooking)

ELISA—enzyme-linked immunosorbent assay.

## Data Availability

No data were used for the research described in the article.

## Supplementary Materials

The following supporting information can be downloaded at: https://www.mdpi.com/article/10.3390/bios15120796/s1, Figure S1. Published systemic reviews and meta-analyses throughout 1995–2022 on the role of CD44-expressed cells in various types of cancer. Breast cancer: [168,169,170,171], Head and neck: [172,173,174,175], Bone: [176,177,178], Gastric: [179,180,181,182,183], Ovarian: [181,182], Pancreatic: [183,184], Renal/bladder: [185,186], Glioma [187,188], Lung cancer: [194,195,196,197], Liver cancer: [198], Esophageal: [199], Colorectal: [156]. Table S1. The role of solCD44 protein in different types of cancer as a diagnostic, prognostic and predictive biomarker. Based on references [1,54,67,69,70,71,79,86,88,90,200]. “CD44 expression on cells” Based on references [31,156,169,170,171,173,177,178,186,201,202,203,204,205,206,207,208,209,210].

## Abbreviations

solCD44	Soluble CD44 protein in serum (circulatory), saliva, urine, or other biological fluids (general idea) or total soluble CD44 (standard and isoforms)
solCD44std	Standard form of soluble CD44 (wild type) without variable exons (exons 1–5, 16–20)
solCD44v1-v10	Soluble variants (isoforms) of CD44 (v1–v10)
Hyaluronic Acid (HA)	Main extracellular ligand that binds CD44 for adhesion and signaling; also used as a ligand in biosensors
CD44 shedding	Proteolytic cleavage releasing soluble CD44, driven by ADAM10/17 and MMP14
