# Supplementary material for: Digging into the Solubility Factor in Cancer Diagnosis: A Case of Soluble CD44 Protein
Source: Biosensors (Basel). 2025 Dec 4;15(12):796. doi: 10.3390/bios15120796 (PMC12731085; doi:10.3390/bios15120796)
Supplement: Supplementary file 1 [file biosensors-15-00796-s001.zip › biosensors-3961297-supplementary.pdf]

Supplementary data

Digging into the solubility factor in cancer diagnosis: a case of soluble CD44 protein

Short running title**: The role of soluble CD44 protein in cancer diagnosis**

Zhuldyz Myrkhiyeva[1,2], Marzhan Nurlankyzy[1,3], Kulzhan Berikkhanova[1,4], Zhanas Baimagambet[3,4], Aidana Bissen[5], Nurzhan Bikhanov[4], Christabel K.L. Tan[6], Daniele Tosi[1,5], Zhannat Ashikbayeva[1,2*], Aliya Bekmurzayeva[1*]

1 Laboratory of Biosensors and Bioinstruments, Center for Life Sciences, National Laboratory Astana,

Nazarbayev University, Astana 010000, Kazakhstan; zhuldyz.myrkhiyeva@nu.edu.kz (Z.M.); marzhan.nurlankyzy@nu.edu.kz (M.N.); kberikkhanova@nu.edu.kz (K.B.); daniele.tosi@nu.edu.kz (D.T.)

2 School of Sciences and Humanities, Nazarbayev University, Astana 010000, Kazakhstan

3 School of Medicine, Nazarbayev University, Astana 010000, Kazakhstan; zhanas.baimagambet@nu.edu.kz

4 University Medical Center, Nazarbayev University, Astana 010000, Kazakhstan; nbikhanov@mail.ru

5 School of Engineering and Digital Sciences, Nazarbayev University, Astana 010000, Kazakhstan;

aidana.bissen@nu.edu.kz

6 School of Physics, Engineering and Computer Science, College Lane, University of Hertfordshire,

Hatfield AL10 9AB, UK; c.k.l.tan@herts.ac.uk

* Correspondence: zhashikbayeva@nu.edu.kz (Z.A.); abekmurzayeva@nu.edu.kz (A.B.)

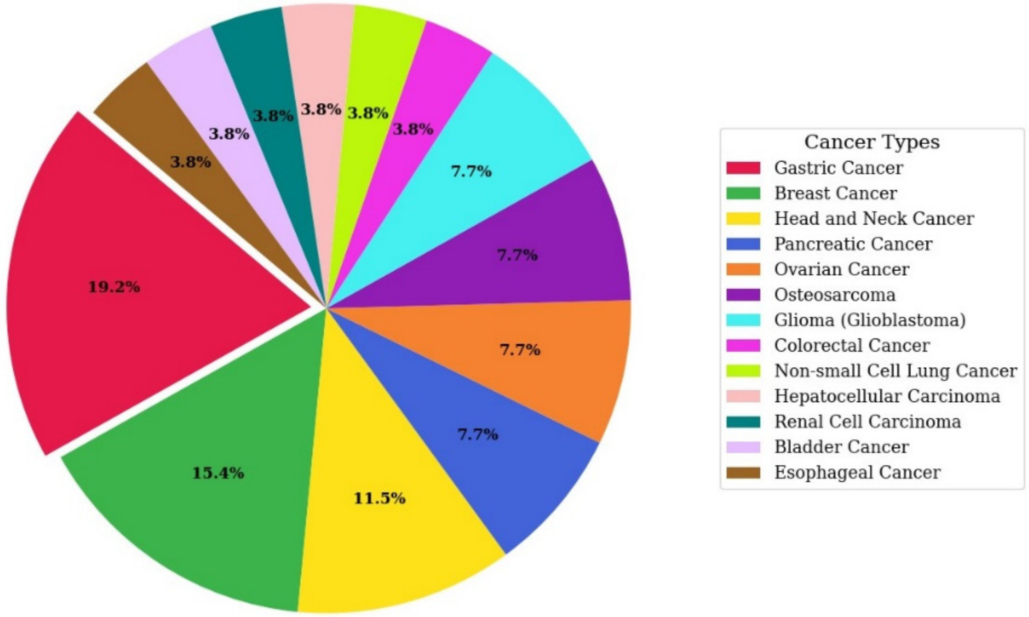

Figure S1. Published systemic reviews and meta-analyses throughout 1995-2022 on the role of CD44-expressed cells in various types of cancer. Breast cancer: [1-4], Head and neck: [5-8], Bone: [9-11], Gastric: [12-16], Ovarian: [17,18], Pancreatic: [19,20], Renal/bladder: [21,22], Glioma [23,24], Lung cancer: [25], Liver cancer: [26], Esophageal: [27], Colorectal: [28].

Supplementary data

Table S1. The role of solCD44 protein in different types of cancer as a diagnostic, prognostic and predictive biomarker. Based on references [29-39]

**Cancer type**<br><br>**(Year of study)**	**Cohort**	**CD44 isoform detected/Method of detection**	**Detected in which biological fluid**	**Main findings/Comments**	**Ref**
HNSCC<br><br>(2007)	Study group: 102 patients with HNSSC<br><br>Control: 69 patients with benign diseases of upper aerodigestive tract	solCD44std<br><br>ELISA assay (Bender MedSystems)	Oral rinse	Sensitivity: 62% to 70%<br>Specificity: 75% to 88%<br><br>solCD44std in saliva 24.4+32.0 ng/mL for HNSCC patients and 9.9+16.1 ng/mL in controls.<br><br>Distinguishes cancer from benign disease with high specificity	[29]
Cervical cancer<br><br>(2014)	Study group:50 cervical cancer patients<br><br>Control: 50 premalignant cases (suspected cases)	solCD44std<br><br>ELISA assay (Bender MedSystems)	Serum	- The threshold value was set as 633.11 ng/ml<br>- Detect and differentiates cancer from premalignant cases with 62.6 % sensitivity	[30]
Acute leukemias and myelodysplastic syndromes<br><br>(1998)	Study group: 162 patients with acute leukemia, myelodysplastic syndrome, chronic myeloid leukemia<br><br>Control: 51 healthy volunteers	solCD44std<br><br>Custom-made ELISA	Serum	- In acute leukemia the serum level of sCD44 decreased after treatment<br>-Might be a valuable prognostic marker for hematological neoplasias	[31]
Breast cancer<br><br>(1995)	Study group: 88 patients	solCD44std<br>solCD44v6<br><br>ELISA	Serum	Not a full-length article (poster)<br><br>solCD44std, solCD44v6:<br>- increased in mts but not in non-mts and healthy<br><br>CD44v6:<br>- level correlates with number of mts sites<br>- correlates with hormone- and chemotherapy	[32]
Breast cancer<br><br>(1999)	Study group: 100 invasive breast carcinoma<br><br>Control: 20 healthy people	solCD44std<br>solCD44v6<br><br>ELISA (Bender MedSystems)	Serum	solCD44std: no significant elevation<br><br>Preoperative solCD44v6 is closely related to distant metastases and TNM staging	[33]

Supplementary data

Cancer type<br><br>(Year of study)	Cohort	CD44 isoform detected/Method of detection	Detected in which biological fluid	Main findings/Comments	Ref
Breast cancer<br><br>(2011)	Study group: 110 patients<br><br>Control:	All isoforms<br><br>ELISA (Abnova)	Serum	Not a randomized or a large-scale prospective study<br><br>- Significant predictor of overall survival in HER2 breast cancer<br>- No association with overall survival in HER2-negative breast cancer<br>- No predictive value after neoadjuvant therapy<br>- Higher conc. in liver metastasis than in other organs	[34]
Breast cancer<br><br>(2018)	Study group: 140 patients stage I-III undergoing mastectomy or breast conserving surgery	All isoforms<br><br>ELISA (Abnova)	Serum	Retrospective study<br><br>- An independent factor for PFS and OS<br>- Higher concentration in triple negative subtype than in luminal	[35]
Breast cancer<br><br>(2008)	Study group: 82 pre-operative primary BC without distant metastasis<br><br>Control: age-matched healthy people	solCD44std<br>solCD44v5<br>solCD44v6<br><br>ELISA<br>(Bender MedSystems)	Serum	solCD44v6:<br>- Increased levels (>75-pecentile) associated with larger tumor size and lymph node mts<br>- Age dependent increase in control group<br>- Might not be suitable as screening marker for breast cancer	[36]
Breast cancer<br><br>(2001)	Study group: 59 patients – metastasis receiving 2nd line hormone- or chemotherapy<br><br>Control: 46 patients with no recurrent disease	solCD44std<br>solCD44v6	Serum	- Similarity in the concentration levels for both proteins for no metastasis patients and healthy control group<br>- Significant correlation between serum solCD44v6 concentrations and the number of metastasized organs, serum lactate dehydrogenase concentrations, tumor grading and the presence of liver metastasis.<br>- Higher CD44v6 in non-responders to second line hormone- and chemotherapy<br>- Unfavorable response to therapy: 250 ng/ml of solCD44v6 and liver metastasis	[37]
HNSCC<br><br>(2010)	Study group: 112 hospitalized patients with oral and maxillofacial malignancy	solCD44v6	Serum and saliva	- Showed a significant association with radiotherapy response in patients with early-stage laryngeal cancer	[38]

Supplementary data

Cancer type<br><br>(Year of study)	Cohort	CD44 isoform detected/Method of detection	Detected in which biological fluid	Main findings/Comments	Ref
	Control: 28 healthy people				
Castrate-resistant prostate cancer (CRPC)<br><br>(2021)	Study group: 66 Castrate-resistant prostate cancer	solCD44 (all forms?), and other proteins: MET, GSN, IL13RA2 and LNPEP<br><br>ELISA (DuoSet; R&D Systems)	Serum	- Independently associated with PFS<br>- May help identify docetaxel-resistant patients<br>- Could be used to help optimize clinical decision-making for therapy (type and time) for metastatic CRPC patients	[39]

HNSCC - head and neck squamous cell carcinoma; mts – metastasis; PFS – progression-free survival; OS – overall survival.

Supplementary data

CD44 expression on cells

The expression of certain CD44 isoforms is correlated with the corresponding type of cancer [40]. Tumor progression and metastasis are associated mainly with the variant isoforms CD44v6 and CD44v9, and normal stratified squamous epithelia, including the epidermis and the lining of the oral cavity, also express CD44 isoforms [41]. Human squamous cell tissue is the only healthy epithelium that physiologically expresses CD44v6; thus, downregulation and loss of specific isoforms can be considered pathological alterations in squamous cell carcinoma of the head and neck [42].

CD44 is implicated in epithelial-to-mesenchymal transition (EMT), a key process in cancer metastasis. This transition is regulated by interactions with proteins such as epithelial splicing regulatory protein 1 (ESRP1) and Snail. Metastasis begins when tumor cells detach from the primary tumor, invade the surrounding extracellular matrix (ECM), and progress through a series of complex steps. Cell surface adhesion molecules are crucial in this process because they mediate cell–environment interactions. CD44 is a primary receptor for hyaluronic acid (HA), a key component of the ECM. It plays an essential role in maintaining tissue homeostasis, preserving biomechanical integrity, and promoting cell migration [43].

In addition to HA, CD44 interacts with other extracellular components, including osteopontin, collagens, fibronectin, and soluble matrix metalloproteinases (MMPs), such as MMP2, MMP7, and MMP9 [44]. These interactions contribute to the dynamic remodeling of the tumor microenvironment. In cancer stem cells (CSCs), high CD44 expression drives tumor progression, therapy resistance, and metastasis by activating signaling pathways such as the PI3K/AKT and Ras-MAPK pathways. The binding of HA to CD44 is particularly critical, as it supports CSC survival, promotes EMT, and fosters aggressive tumor behavior, underscoring the essential role of CD44 in the tumor microenvironment and metastatic progression [45].

Some cancer tissues are well known to express CD44 on their surface. A meta-analysis (involving 12 studies, 898 cases and 1,853 controls) investigating the prognostic role of $CD44^{+}/CD24^{-/low}$ or $ALDH1^{+}$ in breast cancer revealed that these cells were significantly associated with poor overall survival [2]. Another meta-analysis involving breast cancer patients revealed that isoform 6 is strongly associated with lymph node metastasis, histological grade, and poor prognosis. This was especially evident in Asian patients [4]. The results of another meta-analysis suggested that CD44+ T cells, together with the $CD24^{-}$ phenotype, are not reliable markers for the following parameters: tumor size, metastasis to lymph nodes, and distant organs in breast cancer patients. On the other hand, the phenotype is useful for disease-free survival and overall survival [3].

A systematic review and meta-analysis covering 48 studies on colorectal cancer (only 3 studies on solCD44 detected by enzyme-linked immunosorbent assay (ELISA)) revealed that CD44 expression was associated with poor overall survival. It has also been concluded that the overexpression of CD44 could be used as a prognostic biomarker in colorectal cancer for the prediction of poor differentiation and metastasis to lymph nodes and distant organs [28].

A previous study identified CD44v8-10 as a key marker for gastric CSCs and revealed its essential role in tumor initiation [46]. An analysis of gastric tumor and adjacent normal tissue samples revealed that CD44v8-10 was the predominant CD44 variant in gastric cancer cells and was significantly enriched in tumor tissues. Functional assays validated its role as a CSC marker, as CD133 failed to be used to isolate these cells. Silencing CD44 reduced the tumor-initiating ability of gastric cancer cells, an effect restored only with the reintroduction of CD44v8-10, not standard CD44. Furthermore, CD44v8-10 enhanced tumor initiation in immunocompromised models, likely through its role in oxidative stress defense, while exhibiting minimal expression in normal tissues. These findings establish CD44v8-10 as a potential therapeutic target for gastric cancer.

CD44 expressed on cells is a promising therapeutic and prognostic biomarker in pancreatic cancer. It is associated with metastasis to distant organs and is an aggressive form of cancer [19]. A meta-analysis based on tissue expression of CD44 (not serum levels) revealed that it is an efficient prognostic factor in pancreatic cancer [19]. Another meta-analysis also reached this conclusion [20]. Additionally, overexpression of CD44 is significantly correlated with lymph node metastasis, vascular invasion, and a poor five-year overall survival rate.

In a clinical study of CD44 levels in cervical cancer patients, patients with tumors presented higher serum solCD44v6 levels than patients without tumors. However, no significant correlation was observed between the serum level of solCD44std or solCD44v5 and tumor status [47]. These studies support that the solCD44v6 level can be used as a potential biomarker for the identification of cancer relapse.

Head and neck squamous cell carcinoma (HNSCC) is considered a destructive type of cancer because it is often identified late, and 90% of HNSCC tumors are aggressive and recurrent [48]. A systematic review and meta-analysis did not find a clear connection between CD44 expression in tissues and oral cancer [6]. As a marker of CSCs, their potential is limited. The relationships between clinical features and CD44 expression were not significant. However, in the case of

laryngeal and pharyngolaryngeal cancers, the expression of all forms and solCD44v6 was associated with a poorer five-year overall survival rate. CD44 is overexpressed in many types of sarcomas and plays important roles in tumor development, metastasis, and drug resistance [49]. Meta-analysis revealed an association between tissue-expressed solCD44v6 and osteosarcoma and that it can be used as a diagnostic marker [10].

To elucidate the role of CD44 in glioblastoma, two approaches have been used: knocking out the CD44 gene and supplementing with HA [50], the findings of which suggest that CD44 is not a good CSC marker for glioblastoma cells. In contrast, CD44 is more highly expressed in the invasive rim of the tumor, indicating that it has a more important role in cancer invasion and migration. A meta-analysis of tissue-expressed CD44v6 in lung cancer revealed that its overexpression was correlated with tumor differentiation, cancer histological type, metastasis to the lymph node, and TNM (tumor, node, metastasis) stage, with no association with the size of the tumor [51].

Together with having increased surface expression, melanoma cells also have an increased level of CD44 shedding. An earlier study in 2000 on melanoma suggested that the serum CD44 level was significantly lower in cancer patients than in normal individuals [52]. However, the level of serum CD44 was not associated with the stage of melanoma. In contrast, no difference in the serum levels of two other adhesion molecules, P-selectin and intercellular adhesion molecule-1, was detected.

The prevalence of oral squamous cell carcinoma (OSCC) is projected to rise in the coming decades, highlighting the need for effective diagnostic tools. CD44, a common CSC marker, has shown promise as a biomarker for early OSCC detection. Immunohistochemistry (IHC) analysis of lateroventral tongue biopsy samples revealed greater CD44 expression in OSCC epithelial samples than in erosive lichen planus and oral dysplastic lesions. Combining CD44 with other biomarkers could enhance the early diagnosis of OSCC, although larger studies with standardized methods are needed to confirm its utility and improve reproducibility [53].

References

1. Wang, Z.; Wang, Q.Q.; Wang, Q.; Wang, Y.P.; Chen, J. Prognostic significance of CD24 and CD44 in breast cancer: a meta-analysis. *International Journal of Biological Markers* **2017**, *32*, E75-E82, doi:10.5301/jbm.5000224.
2. Zhou, L.; Jiang, Y.; Yan, T.; Di, G.; Shen, Z.; Shao, Z.; Lu, J. The prognostic role of cancer stem cells in breast cancer: a meta-analysis of published literatures. *Breast Cancer Research and Treatment* **2010**, *122*, 795-801, doi:10.1007/s10549-010-0999-4.
3. Gu, J.J.; Chen, D.D.; Li, Z.Q.; Yang, Y.L.; Ma, Z.M.; Huang, G.H. Prognosis assessment of CD44(+)/CD24(-) in breast cancer patients: a systematic review and meta-analysis. *Archives of Gynecology and Obstetrics* **2022**, *306*, 1147-1160, doi:10.1007/s00404-022-06402-w.
4. Qiao, G.L.; Song, L.N.; Deng, Z.F.; Chen, Y.; Ma, L.J. Prognostic value of CD44v6 expression in breast cancer: a meta-analysis. *Oncotargets and Therapy* **2018**, *11*, 5451-5457, doi:10.2147/ott.s156101.
5. Chai, L.; Liu, H.Y.; Zhang, Z.L.; Wang, F.; Wang, Q.Y.; Zhou, S.H.; Wang, S.Q. CD44 Expression Is Predictive of Poor Prognosis in Pharyngolaryngeal Cancer: Systematic Review and Meta-Analysis. *Tohoku Journal of Experimental Medicine* **2014**, *232*, 9-19, doi:10.1620/tjem.232.9.
6. Chen, J.Q.; Zhou, J.D.; Lu, J.; Xiong, H.; Shi, X.L.; Gong, L. Significance of CD44 expression in head and neck cancer: a systemic review and meta-analysis. *Bmc Cancer* **2014**, *14*, doi:10.1186/1471-2407-14-15.
7. Chen, Y.; Sun, W.W.; Li, P.H.; Tang, Q.; Liu, Y.; Xue, Q.; Xu, X.Y. Clinicopathological significance of cancer stem cell markers in nasopharyngeal carcinoma: a meta-analysis. *International Journal of Clinical and Experimental Medicine* **2017**, *10*, 6138-6147.
8. Krishnan, R.; Pandiar, D.; Ramani, P.; Ramalingam, K.; Jayaraman, S. Utility of CD44/CD24 in the Outcome and Prognosis of Oral Squamous Cell Carcinoma: A Systematic Review. *CUREUS JOURNAL OF MEDICAL SCIENCE* **2023**, *15*, doi:10.7759/cureus.42899.
9. Zhang, Y.; Ding, C.; Wang, J.; Sun, G.; Cao, Y.; Xu, L.; Zhou, L.; Chen, X. Prognostic significance of CD44V6 expression in osteosarcoma: a meta-analysis. *JOURNAL OF ORTHOPAEDIC SURGERY AND RESEARCH* **2015**, *10*, doi:10.1186/s13018-015-0328-z.

10. Zhang, Y.Y.; Lun, L.M.; Zhu, B.Z.; Wang, Q.; Ding, C.M.; Hu, Y.L.; Huang, W.L.; Zhou, L.; Chen, X.; Huang, H. Diagnostic accuracy of CD44V6 for osteosarcoma: a meta-analysis. *Journal of Orthopaedic Surgery and Research* **2016**, *11*, doi:10.1186/s13018-016-0470-2.
11. Liu, Y.; Wu, Y.W.; Gu, S.J.; Sun, Z.Z.; Rui, Y.J.; Wang, J.B.; Lu, Y.; Li, H.F.; Xu, K.L.; Sheng, P. Prognostic role of CD44 expression in osteosarcoma: evidence from six studies. *Diagnostic Pathology* **2014**, *9*, doi:10.1186/1746-1596-9-140.
12. Wang, W.; Dong, L.P.; Zhang, N.; Zhao, C.H. Role of cancer stem cell marker CD44 in gastric cancer: a meta-analysis. *International Journal of Clinical and Experimental Medicine* **2014**, *7*, 5059-5066.
13. Wu, Y.; Li, Z.; Zhang, C.L.; Yu, K.; Teng, Z.; Zheng, G.L.; Wang, S.; Liu, Y.P.; Cui, L.; Yu, X.S. CD44 family proteins in gastric cancer: a meta-analysis and narrative review. *International Journal of Clinical and Experimental Medicine* **2015**, *8*, 3595-U1695.
14. Chen, Y.S.; Fu, Z.Y.; Xu, S.J.; Xu, Y.; Xu, P.F. The prognostic value of CD44 expression in gastric cancer: A meta-Analysis. *Biomedicine & Pharmacotherapy* **2014**, *68*, 693-697, doi:10.1016/j.biopha.2014.08.001.
15. Fang, M.; Wu, J.R.; Lai, X.; Ai, H.Y.; Tao, Y.F.; Zhu, B.; Huang, L.S. CD44 and CD44v6 are Correlated with Gastric Cancer Progression and Poor Patient Prognosis: Evidence from 42 Studies. *Cellular Physiology and Biochemistry* **2016**, *40*, 567-578, doi:10.1159/000452570.
16. Gao, S.; Zhao, Z.Y.; Wu, R.; Zhang, Y.; Zhang, Z.Y. Prognostic value of long noncoding RNAs in gastric cancer: a meta-analysis. *Oncotargets and Therapy* **2018**, *11*, 4877-4891, doi:10.2147/ott.s169823.
17. Shi, Y.Y.; Jiang, H. Prognostic role of the cancer stem cell marker CD44 in ovarian cancer: a meta-analysis. *Genetics and Molecular Research* **2016**, *15*, doi:10.4238/gmr.15038325.
18. Zhao, L.Y.; Gu, C.L.; Huang, K.; Zhang, Z.; Ye, M.X.; Fan, W.S.; Han, W.D.; Meng, Y.G. The prognostic value and clinicopathological significance of CD44 expression in ovarian cancer: a meta-analysis. *Archives of Gynecology and Obstetrics* **2016**, *294*, 1019-1029, doi:10.1007/s00404-016-4137-3.
19. Huang, H.C.; Zhang, Z.; Huang, D.B.; Zhang, X.Z. CD44 expression and its clinical significance in pancreatic cancer: a meta-analysis. *International Journal of Clinical and Experimental Medicine* **2018**, *11*, 5350-5358.
20. Liu, Y.J.; Wu, T.; Lu, D.; Zhen, J.T.; Zhang, L. CD44 overexpression related to lymph node metastasis and poor prognosis of pancreatic cancer. *International Journal of Biological Markers* **2018**, *33*, 308-313, doi:10.1177/1724600817746951.
21. Li, X.T.; Ma, X.; Chen, L.Y.; Gu, L.Y.; Zhang, Y.; Zhang, F.; Ouyang, Y.; Gao, Y.; Huang, Q.B.; Zhang, X. Prognostic value of CD44 expression in renal cell carcinoma: a systematic review and meta-analysis. *Scientific Reports* **2015**, *5*, doi:10.1038/srep13157.
22. Hu, Y.; Zhang, Y.R.; Gao, J.L.; Lian, X.; Wang, Y.T. The clinicopathological and prognostic value of CD44 expression in bladder cancer: a study based on meta-analysis and TCGA data. *Bioengineered* **2020**, *11*, 572-581, doi:10.1080/21655979.2020.1765500.
23. Wu, G.; Song, X.H.; Liu, J.; Li, S.Z.; Gao, W.Q.; Qiu, M.X.; Yang, C.J.; Ma, Y.M.; Chen, Y.H. Expression of CD44 and the survival in glioma: a meta-analysis. *Bioscience Reports* **2020**, *40*, doi:10.1042/bsr20200520.
24. Hou, C.X.; Ishi, Y.; Motegi, H.; Okamoto, M.; Ou, Y.F.; Chen, J.W.; Yamaguchi, S. Overexpression of CD44 is associated with a poor prognosis in grade II/III gliomas. *Journal of Neuro-Oncology* **2019**, *145*, 201-210, doi:10.1007/s11060-019-03288-8.
25. Jiang, H.; Zhao, W.; Shao, W. Prognostic value of CD44 and CD44v6 expression in patients with non-small cell lung cancer: meta-analysis. *Tumor Biology* **2014**, *35*, 7383-7389, doi:10.1007/s13277-014-2150-3.
26. Luo, Y.K.; Tan, Y. Prognostic value of CD44 expression in patients with hepatocellular carcinoma: meta-analysis. *Cancer Cell International* **2016**, *16*, doi:10.1186/s12935-016-0325-2.
27. Al-mosawi, A.K.M.; Cheshomi, H.; Hosseinzadeh, A.; Matin, M.M. Prognostic and Clinical Value of CD44 and CD133 in Esophageal Cancer: A Systematic Review and Meta-analysis. *Iranian Journal of Allergy Asthma and Immunology* **2020**, *19*, 105-116, doi:10.18502/ijaai.v19i2.2756.

28. Wang, Z.P.; Tang, Y.F.; Xie, L.; Huang, A.P.; Xue, C.C.; Gu, Z.; Wang, K.Q.; Zong, S.Q. The Prognostic and Clinical Value of CD44 in Colorectal Cancer: A Meta-Analysis. *Frontiers in Oncology* **2019**, *9*, doi:10.3389/fonc.2019.00309.
29. Franzmann, E.J.; Reategui, E.P.; Pedroso, F.; Pernas, F.G.; Karakullukcu, B.M.; Carraway, K.L.; Hamilton, K.; Singal, R.; Goodwinl, W.J. Soluble CD44 is a potential marker for the early detection of head and neck cancer. *Cancer Epidemiology Biomarkers & Prevention* **2007**, *16*, 1348-1355, doi:10.1158/1055-9965.epi-06-0011.
30. Dasari, S.; Rajendra, W.; Valluru, L. Evaluation of soluble CD44 protein marker to distinguish the premalignant and malignant carcinoma cases in cervical cancer patients. *Medical Oncology* **2014**, *31*, doi:10.1007/s12032-014-0139-9.
31. Nasu, H.; Hibi, N.; Ohyashiki, J.H.; Hara, A.; Kubono, K.; Tsukada, Y.; Ando, K.; Iwama, H.; Hayashi, S.; Yahata, N.; et al. Serum soluble CD44 levels for monitoring disease states in acute leukemia and myelodysplastic syndromes. *International Journal of Oncology* **1998**, *13*, 525-530.
32. Classen, S.D.; Wolf, H.; Eiermann, W.; Kopp, R.; Rieskamp, G.; Schildberg, F.W.; Wilmanns, W. Soluble CD44 standard and v6 in serum of breast cancer patients: An indicator for therapy response. *European Journal of Cancer* **1995**, *31A*, 659-659.
33. Sheen-Chen, S.M.; Chen, W.J.; Eng, H.L.; Sheen, C.C.; Chou, F.F.; Cheng, Y.F. Evaluation of the prognostic value of serum soluble CD 44 in patients with breast cancer. *Cancer Investigation* **1999**, *17*, 581-585, doi:10.3109/07357909909032843.
34. Baek, J.M.; Jin, Q.R.; Ensor, J.; Boulbes, D.R.; Esteva, F.J. Serum CD44 levels and overall survival in patients with HER2-positive breast cancer. *Breast Cancer Research and Treatment* **2011**, *130*, 1029-1036, doi:10.1007/s10549-011-1691-z.
35. Kong, Y.A.; Lyu, N.; Wu, J.L.; Tang, H.L.; Xie, X.H.; Yang, L.; Li, X.; Wei, W.D.; Xie, X.M. Breast cancer stem cell markers CD44 and ALDH1A1 in serum: distribution and prognostic value in patients with primary breast cancer. *Journal of Cancer* **2018**, *9*, 3728-3735, doi:10.7150/jca.28032.
36. Mayer, S.; zur Hausen, A.; Watermann, D.O.; Stamm, S.; Jager, M.; Gitsch, G.; Stickeler, E. Increased soluble CD44 concentrations are associated with larger tumor size and lymph node metastasis in breast cancer patients. *Journal of Cancer Research and Clinical Oncology* **2008**, *134*, 1229-1235, doi:10.1007/s00432-008-0397-z.
37. Kopp, R.; Classen, S.; Wolf, H.; Gholam, P.; Possinger, K.; Eiermann, W.; Wilmanns, W. Predictive relevance of soluble CD44v6 serum levels for the responsiveness to second line hormone- or chemotherapy in patients with metastatic breast cancer. *Anticancer Research* **2001**, *21*, 2995-3000.
38. de Jong, M.C.; Pramana, J.; van der Wal, J.E.; Lacko, M.; Peutz-Kootstra, C.J.; de Jong, J.M.; Takes, R.P.; Kaanders, J.H.; van der Laan, B.F.; Wachters, J.; et al. CD44 Expression Predicts Local Recurrence after Radiotherapy in Larynx Cancer. *Clinical Cancer Research* **2010**, *16*, 5329-5338, doi:10.1158/1078-0432.ccr-10-0799.
39. Keresztes, D.; Csizmarik, A.; Nagy, N.; Modos, O.; Fazekas, T.; Bracht, T.; Sitek, B.; Witzke, K.; Puhr, M.; Sevcenco, S.; et al. Comparative proteome analysis identified CD44 as a possible serum marker for docetaxel resistance in castration-resistant prostate cancer. *Journal of Cellular and Molecular Medicine* **2022**, *26*, 1332-1337, doi:10.1111/jcmm.17141.
40. Yusupov, M.; Privat-Maldonado, A.; Cordeiro, R.M.; Verswyvel, H.; Shaw, P.; Razzokov, J.; Smits, E.; Bogaerts, A. Oxidative damage to hyaluronan-CD44 interactions as an underlying mechanism of action of oxidative stress-inducing cancer therapy. *Redox Biology* **2021**, *43*, doi:10.1016/j.redox.2021.101968.
41. Chang, S.M.; Xing, R.D.; Zhang, F.M.; Duan, Y.Q. Serum soluble CD44v6 levels in patients with oral and maxillofacial malignancy. *Oral Diseases* **2009**, *15*, 570-572, doi:10.1111/j.1601-0825.2009.01591.x.
42. Andratschke, M.; Chaubal, S.; Pauli, C.; Mack, B.; Hagedorn, H.; Wollenberg, B. Soluble CD44v6 is not a sensitive tumor marker in patients with head and neck squamous cell cancer. *Anticancer Research* **2005**, *25*, 2821-2826.

43. Reinke, L.; Xu, Y.; Cheng, C. Snail Represses the Splicing Regulator Epithelial Splicing Regulatory Protein 1 to Promote Epithelial-Mesenchymal Transition. *JOURNAL OF BIOLOGICAL CHEMISTRY* **2012**, *287*, 36435-36442, doi:10.1074/jbc.M112.397125.
44. Wöhner, B.; Li, W.; Hey, S.; Drobny, A.; Werny, L.; Becker-Pauly, C.; Lucius, R.; Zunke, F.; Linder, S.; Arnold, P. Proteolysis of CD44 at the cell surface controls a downstream protease network. *FRONTIERS IN MOLECULAR BIOSCIENCES* **2023**, *10*, doi:10.3389/fmolb.2023.1026810.
45. Sapudom, J.; Ullm, F.; Martin, S.; Kalbitzer, L.; Naab, J.; Moller, S.; Schnabelrauch, M.; Anderegg, U.; Schmidt, S.; Pompe, T. Molecular weight specific impact of soluble and immobilized hyaluronan on CD44 expressing melanoma cells in 3D collagen matrices. *Acta Biomaterialia* **2017**, *50*, 259-270, doi:10.1016/j.actbio.2016.12.026.
46. Lau, W.M.; Teng, E.; Chong, H.S.; Lopez, K.A.P.; Tay, A.Y.L.; Salto-Tellez, M.; Shabbir, A.; So, J.B.Y.; Chan, S.L. CD44v8-10 Is a Cancer-Specific Marker for Gastric Cancer Stem Cells. *Cancer Research* **2014**, *74*, 2630-2641, doi:10.1158/0008-5472.can-13-2309.
47. Kainz, C.; Tempfer, C.; Winkler, S.; Sliutz, G.; Koelbl, H.; Reinthaller, A. Serum CD44 splice variants in cervical cancer patients. *Cancer Letters* **1995**, *90*, 231-234, doi:10.1016/0304-3835(95)03708-5.
48. Kokko, L.L.; Hurme, S.; Maula, S.M.; Alanen, K.; Grénman, R.; Kinnunen, I.; Ventelä, S. Significance of site-specific prognosis of cancer stem cell marker CD44 in head and neck squamous-cell carcinoma. *Oral Oncology* **2011**, *47*, 510-516, doi:10.1016/j.oraloncology.2011.03.026.
49. Fernandez-Tabanera, E.; de Mera, R.; Alonso, J. CD44 In Sarcomas: A Comprehensive Review and Future Perspectives. *Frontiers in Oncology* **2022**, *12*, doi:10.3389/fonc.2022.909450.
50. Wang, H.H.; Liao, C.C.; Chow, N.H.; Huang, L.L.H.; Chuang, J.I.; Wei, K.C.; Shin, J.W. Whether CD44 is an applicable marker for glioma stem cells. *American Journal of Translational Research* **2017**, *9*, 4785-4806.
51. Luo, Z.; Wu, R.R.; Lv, L.; Li, P.; Zhang, L.Y.; Hao, Q.L.; Li, W. Prognostic value of CD44 expression in non-small cell lung cancer: a systematic review. *International Journal of Clinical and Experimental Pathology* **2014**, *7*, 3632-3646.
52. Yasasever, Y.; Tas, F.; Duranyildiz, D.; Camlica, H.; Kurul, S.; Dalay, N. Serum levels of the soluble adhesion molecules in patients with malignant melanoma. *Pathology and Oncology Research* **2000**, *6*, 42-45, doi:10.1007/BF03032657.
53. Bader, A. The Diagnostic Utility of Cancer Stem Cell Marker CD44 in Early Detection of Oral Cancer. University Maryland, Baltimore, 2019.
